# Directed assembly of single-stranded DNA fragments for data storage via protein-free catalytic splint ligation

**DOI:** 10.1093/nar/gkaf582

**Published:** 2025-06-30

**Authors:** Gemma Mendonsa, Sriram Chari, Mengdi Bao, Brett Herdendorf, Anil Reddy

**Affiliations:** Seagate Research Group, Seagate Technology, 1280 Disc Dr, Shakopee, MN 55379, United States; Seagate Research Group, Seagate Technology, 1280 Disc Dr, Shakopee, MN 55379, United States; Seagate Research Group, Seagate Technology, 1280 Disc Dr, Shakopee, MN 55379, United States; Seagate Research Group, Seagate Technology, 1280 Disc Dr, Shakopee, MN 55379, United States; Seagate Research Group, Seagate Technology, 1280 Disc Dr, Shakopee, MN 55379, United States

## Abstract

Oligonucleotides or gene fragments can be ligated in a specified order to create longer DNA assemblies. We present a method where DNA symbols, or oligos designed to encode information for archival data storage, are joined to linker sequences at either end. These linkers dictate the assembly order of the symbols; the order of the symbols can be changed by changing the sequences of the linkers attached to them. Utilizing a ligating DNAzyme as a catalytic splint, we achieve room-temperature, protein-free assembly, offering a cost-effective alternative to traditional enzyme-based ligation methods. We demonstrate this technique by assembling three different five-symbol constructs, with the order of the symbols determined by their linking ends. This linker directed assembly technique allows data-encoding symbols to be assembled in any desired order. Furthermore, the DNAzyme-based assembly method is versatile and can be applied to various DNA assembly applications, particularly where cost-effectiveness and efficient room-temperature ligation are required.

## Graphical Abstract

**Graphical Abstract:**
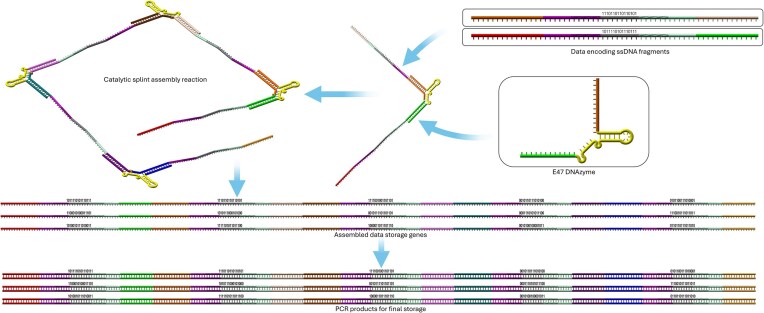

## Introduction

### The case for DNA data storage

The growth of data generation is projected to reach 180 zettabytes (180 × 10^21^ bytes) annually by 2025 [1]. The majority of enterprise data being generated are classified as “cold/archival,” i.e. data that need to be stored, but do not need to be accessed frequently, if ever (https://www.twistbioscience.com/resources/white-paper/escalating-challenge-preserving-enterprise-data). Archiving digital data necessitates significant physical space and electrical power, depending on the storage technology employed. In addition, most storage systems, due to media durability or the obsolescence of writer/reader hardware, or of data formats or protocols, require periodic migration of archived data to fresh media [2]. To be able to archive more of the newly generated data, paradigm-shifting new storage methods are a necessity.

DNA, nature’s medium for genomic data storage, offers an unprecedented level of volumetric storage density. A single gram of DNA can theoretically store up to 215 petabytes (1 PB = 10^15^ bytes) [3], which is many orders of magnitude greater than solid-state drives, hard disk drives , and tape media [4]. Furthermore, DNA has a half-life of ∼500 years and can be replicated with relative ease, thereby reducing the need for new storage media solely for the purpose of refreshing previously archived data [2]. DNA is an excellent candidate for so-called glacial digital archives, i.e. data that can be stored offline, and possibly remotely, with only very rare access expected, if any [5–10]. For such archives, there is a willingness to accept trade-offs in read/write times in favor of volumetric storage density, low maintenance, and low energy consumption [10–12].

For these reasons, DNA data storage has generated substantial interest from both caretakers of large data archives and researchers in both academia and industry seeking to transition DNA data storage from a research concept to a commercially viable solution. In their white paper, the DNA Data Storage Alliance highlights the “Data Storage Pipeline,” which is comprised of coding (from bits to nucleotides), synthesis, storage, retrieval, sequencing, and decoding [5]. Advances in all sub-systems are needed to commercialize DNA data storage; our work focuses on the synthesis/write process, which is generally believed to be the limiting step of the pipeline [13].

### Current limitations in DNA data writing

DNA synthesis typically relies on phosphoramidite chemistry, a powerful and well-established technology for synthesizing short DNA strands, one nucleotide at a time. There are key limitations that must be considered, however, when operating at DNA data storage scales; these include the synthesis rate, reagent volumes, environmental considerations, and cost. It takes around 4–10 min per base to write a single nucleotide during phosphoramidite synthesis [14]. Phosphoramidite-based methods also require large volumes of flammable, oxidative, and corrosive solvents [10], which could limit molecular storage adoption by data hyperscalers [15]. Estimated write costs for DNA data storage using phosphoramidite chemistry are $70M USD per TB at the low end [13], rendering DNA storage to be cost-prohibitive without substantial changes to the dominant synthesis approaches. Enzymatic synthesis is a promising technology that may be faster and use less hazardous reagents than phosphoramidite synthesis [16, 17], but it remains in the research phase and faces challenges with parallelization and scalability [13].

### Proposed approach to address limitations in DNA data writing

Oligo assembly-based approaches have been proposed to increase the write speed in DNA data storage. Yan *et al.* [18] introduced an enzymatic ligation technique for synthesizing storage genes based on composite motifs as building blocks. A dual-library system of “symbols” and “linkers” has also been proposed [19, 20]. The “symbols” carry the data payload and the “linkers” are short oligos that help concatenate these symbols in the correct order. The libraries work together to create long “genes” with symbols in the correct order, representing the data. The first aim of this work is to demonstrate this dual-library assembly approach by assembling symbols in specified orders to create several different data storage genes.

In conventional DNA synthesis, nucleotides are sequentially attached one at a time, requiring *N* synthesis steps to construct a strand of *N* nucleotides. Assuming each nucleotide can store 2 bits of information, this results in a linear relationship between synthesis time and storage capacity. Motif-by-motif construction increases the data synthesized per reaction step by a factor corresponding to motif length. Our symbol + linker approach leverages a geometric progression-based assembly strategy to accelerate the synthesis process.

**Figure 1.**
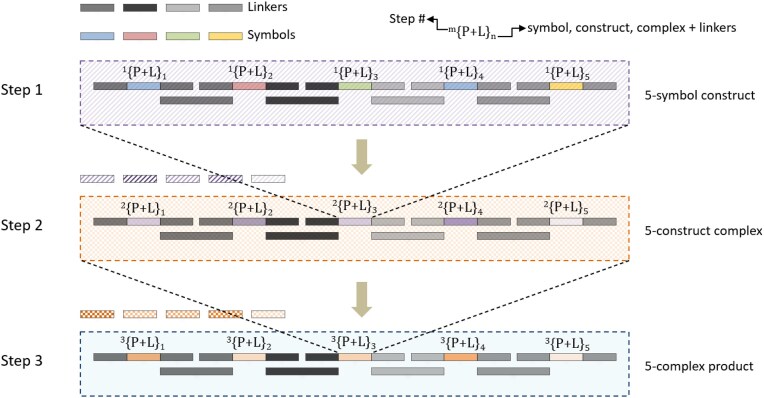
Each solid block corresponds to an eight nucleotide long symbol. Different pastel shades represent different symbols. Solid blocks with different shades of black represent linkers. (P+L)*n* indicates a symbol/construct/complex with attached linkers, and *m* indicates step number. In Step 1, five symbols with linkers attached to their ends are ligated together to form a “five-symbol” construct. In Step 2, each of these five-symbol constructs is brought together to form a “five-construct” complex. Each complex effectively contains 25 symbols. In Step 3, each of the five-construct complexes is brought together to form a “five-complex” product. Each product of Step 3 effectively contains 125 symbols.

Each symbol consists of eight nucleotides, encoding 16 bits of information. In the first reaction step, a symbol and two linkers combine. In the second step, five symbol–linker complexes merge, forming a strand that can store 80 bits of information. This process can theoretically continue for multiple additional assemblies to increase growth geometrically. For instance, after three five-piece assembly steps, the assembled strand holds 125 symbols (2000 bits of stored information) (Fig. 1). This advancement paves the way for faster ultra-high-density molecular data archiving.

Synthesizing oligos in bulk is a much cheaper process than synthesizing each individual data storage gene from scratch [18], but the high cost of the ligase used to ligate the oligos still results in inordinately expensive DNA assembly at scales needed for data storage. For example, using the current pricing of DNA assembly reagents from New England Biolabs (NEB), it is estimated that the total cost of the reagents needed for ligating oligos together using standard T4 ligase is $1.36 for a 20 µl reaction (https://www.neb.com/en-us/products/m0202-t4-dna-ligase), while the cost of the ligase is $1.35 (https://www.neb.com/en-us/products/b0202-t4-dna-ligase-reaction-buffer), making up the majority of the cost. While the costs of protein-based enzymes are manageable at benchtop-scale experiments, when the reagent volumes are scaled up to data-storage-level volumes the cost of these methods remains prohibitively high.

To address this drawback, the second aim of this work is to introduce a novel cost-effective DNA assembly method that requires no protein-based enzymes. Our approach involves the use of DNAzymes for concatenating short DNA segments through chemical ligation to build the desired storage gene [21]. The DNAzymes act as catalytic molecular splints, bringing together the ends of DNA strands and catalyzing the formation of phosphodiester bonds. This process eliminates the need for ligase or any other protein-based enzymes during the ligation step [22]. DNAzyme-based ligation uses aqueous solvents, rather than large volumes of flammable or oxidative solvents [13].

We describe here a novel synthesis strategy and assembly chemistry that has the potential to offer the DNA data storage community a means to achieve geometric DNA strand growth, rather than the nucleotide-by-nucleotide synthesis strategy employed by many researchers in this field, in a cost-effective manner with a reduced environmental footprint.

## Materials and methods

### Materials

**Figure 2.**
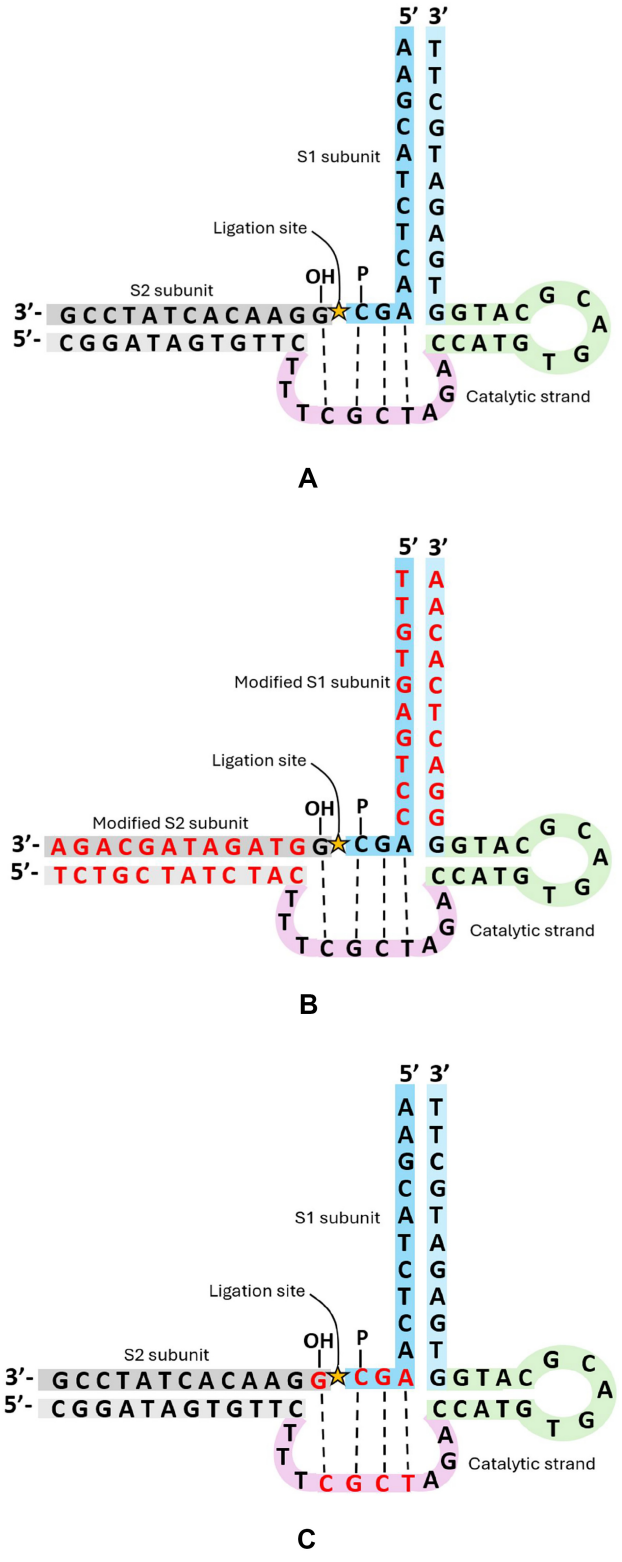
(**A**) The original E47 DNAzyme and subunits as reported by Cuenoud and Szostak [23]. (**B**) E47 with modified substrates and substrate binding arms. (**C**) E47 four-base catalytic motif. The bases in this region are likely directly involved in catalysis. The four bases in this catalytic motif region were mutated one by one to explore the sequence dependence of this region.

The oligos used in the Fig. 2 experiment were synthesized using an H-8 oligo synthesizer by K&A Laboratories according to the manufacturer’s instructions. Size markers used in the gel electrophoresis experiments for five-piece assemblies were synthesized by GENEWIZ from Azenta Life Sciences. Oligos used in five-piece assembly quantification were synthesized by Bioneer. The single-stranded DNA (ssDNA) 50 ladder used in Supplementary Fig. S4 was purchased from GeneBio Systems, Inc. All other oligos and ssDNA ladders were purchased from Integrated DNA Technologies. Phusion PCR kit, dNTPs, Zeba spin columns, TBE buffer, HPLC-grade acetonitrile, PureLink Gel Extraction kit, and 100-bp ladder were purchased from Thermo Scientific. Certified low-range agarose was purchased from Bio-Rad. DeepVent and DeepVent (exo−) polymerases and PCR buffers were purchased from NEB. SD Polymerase was purchased from Bioron. NAP-5 desalting columns were purchased from Cytiva. Sanger Sequencing reagents were purchased from Promega. Imidazole, EDC, ZnCl_2_, HEPES, NaCl, PAGE gel electrophoresis reagents, triethylammonium acetate, and SYBR Green I/II gel stains were purchased from Sigma–Aldrich. Rapid Barcoding Kit and Oxford Nanopore sequencing reagents were purchased from Oxford Nanopore. DNA sequences are available in the Supplementary data (Supplementary Table S1).

### Two-piece DNAzyme activation and ligation

#### S1 subunit activation

Fresh 1 M EDC solution was prepared. A solution of 80 µM S1 subunit, 100, 20, or 5 mM imidazole (pH 6.0), and 100 mM EDC was mixed, vortexed, and incubated on the benchtop at room temperature for 60 min. If a purification step was being performed, the activation solution was desalted with a Zeba 7K MWCO spin column according to manufacturer’s instructions. The DNA amount remaining in the desalted solution was quantified by a NanoDrop OneC. Activated S1 was immediately used in a ligation reaction.

#### Two-piece ligation

A solution of 2 µM S2 subunit, 3 µM catalytic strand, 4 µM activated S1 subunit, 5, 10, or 20 mM imidazole, 100 mM EDC, and 4 mM ZnCl_2_ was prepared in a buffer containing 30 mM HEPES and 300 mM NaCl at pH 7.0. Negative controls had no ZnCl_2_ added. The ligation solutions were incubated at room temperature on the benchtop for 120 min. After the ligation period, a 1/10 volume of 100 mM EDTA (pH 8.0) was added to the ligation solutions.

#### One-step two-piece ligation

A solution of 2 µM S2 subunit, 3 µM catalytic strand, 4 µM activated S1 subunit, and 4 mM ZnCl_2_ was prepared in a buffer containing 30 mM HEPES and 300 mM NaCl at pH 7.0. Negative controls had no ZnCl_2_ added. The ligation solutions were incubated at room temperature on the benchtop for 120 min. After the ligation period, a 1/10 volume of 100 mM EDTA (pH 8.0) was added to the ligation solutions.

#### Two-piece quantification

Standards of the known ligation product were run, and the resulting peak areas plotted versus concentration with JMP. Standard curves, equations, and fits were generated using JMP (Supplementary Fig. S1A and B).

### Three-piece DNAzyme activation and ligation

#### S1 subunit activation

Fresh 1 M EDC solution was prepared. A solution of 80 µM S1 subunit, 20 mM imidazole (pH 6.0), and 100 mM EDC was mixed, vortexed, and incubated on the benchtop at room temperature for 60 min. The activation solution was desalted with a Zeba 7K MWCO spin column according to the manufacturer’s instructions. The DNA amount remaining in the desalted solution was quantified by a NanoDrop OneC. Desalted activated S1 was immediately used in a ligation reaction.

#### Three-piece ligation

A solution of 2 µM S2 subunit, 4 µM catalytic strand A, 4 µM catalytic strand B, 4 µM activated S1 subunit A, 4 µM activated S1 subunit B, and 4 mM ZnCl_2_ was prepared in a buffer containing 30 mM HEPES and 300 mM NaCl at pH 7.0. Negative controls had no ZnCl_2_ added. The ligation solutions were incubated at room temperature on the benchtop for 120 min. After the ligation period, a 1/10 volume of 100 mM EDTA (pH 8.0) was added to the ligation solutions. The full-length ligation products were purified by high-performance liquid chromatography (HPLC) and desalted with NAP-5 columns according to the manufacturer’s instructions. The desalted ligation product was concentrated in a 60°C vacuum centrifuge for 1.5 h. The yield was quantified via HPLC: two different three-piece assemblies with three replicates each were compared to a standard curve of the expected product (Supplementary Fig. S2A and B). The six calculated yields were averaged.

### Five-piece DNAzyme activation and ligation

#### S1 subunit activation

Fresh 1 M EDC solution was prepared. A solution of ∼1 µM of each of four linker–symbol assemblies, 20 mM imidazole (pH 6.0), and 100 mM EDC was mixed, vortexed, and incubated on the benchtop at room temperature for 60 min. The activation solution was not desalted, but immediately used in a ligation reaction.

#### Five-piece ligation

A solution of 0.1 µM S2 subunit, 0.1 µM each of four catalytic strands, and ∼0.4 µM activated S1 subunit mix, and 4 mM ZnCl_2_ was prepared in a buffer containing 30 mM HEPES and 300 mM NaCl at pH 7.0. Negative controls had no ZnCl_2_ added. The ligation solutions were incubated at room temperature on the benchtop for 3 h. After the ligation period, a 1/10 volume of 100 mM EDTA (pH 8.0) was added to the ligation solutions. The full-length ligation products were analyzed and purified by gel electrophoresis.

#### Quantification

To quantify the yield of the five-piece reaction, three-piece assemblies were purchased from Bioneer rather than assembled, so the product would not be lost during purification and the concentration of the reactants would be known precisely. The assembly was performed as above in triplicate, and the products run on a 12% PAGE gel. Standards of the known ligation product were run on the gel; the resulting band intensities were measured with ImageJ and plotted versus concentration with JMP. Standard curves, equations, and fits were generated using JMP (Supplementary Fig. S3A and B).

### Two-component DNAzyme activation and ligation

#### S1 subunit activaton

Fresh 1 M EDC solution was prepared. A solution of ∼1 µM of each of four linker–symbol assemblies, 100 mM imidazole (pH 6.0), and 100 mM EDC was mixed, vortexed, and incubated on the benchtop at room temperature for 60 min. The activation solution was not desalted, but immediately used in a ligation reaction.

#### Two-component assembly

A solution of 8 µM activated S1 subunit, 8 µM catalytic strand, 100 mM imidazole, 100 mM EDC, and 10 mM ZnCl_2_ was prepared in a buffer containing 30 mM HEPES and 300 mM NaCl at pH 7.0. Negative controls had no ZnCl_2_ added. The ligation solutions were incubated at room temperature on the benchtop for 180 min. After the ligation period, a 1/10 volume of 500 mM EDTA (pH 8.0) was added to the ligation solutions. The crude reactions were run on a 12% PAGE gel and imaged with a BioRad GelDoc Go (Supplementary Fig. S4).

### HPLC analysis and purification

HPLC was performed on an Agilent 1100 series HPLC system with DAD detector and fraction collector. The Waters XBridge Oligonucleotide C18 BEH column was heated to 80°C. Mobile phase A consisted of 100 mM triethylammonium acetate (TEAA) buffer (pH 7.0). Mobile phase B consisted of 80 mM TEAA and 20% (v/v) HPLC-grade acetonitrile. A gradient of 60% A/40% B was adjusted to 40.5% A/59.5% B over the course of 7.5 min at 0.6 ml/min. If purification was being performed, ligation products were collected by a fraction collector and concentrated in a 60°C vacuum centrifuge for 30 min.

### Gel electrophoresis (PAGE)

A freshly prepared 1.6% (m/v) solution of ammonium persulfate (APS) was prepared in ultrapure water. A gel solution of 12% acrylamide and 8 M urea was prepared in 1× TBE. 2.2 ml of the APS solution was added for every 50 ml of gel. 33.3 µl of TEMED was added for every 50 ml of gel, and the gel was immediately cast in 20 cm × 20 cm plates separated by 1.5 mm spacers. The gel polymerized for 90 min, and then was loaded into a Bio-Rad Protean Xi electrophoresis core. Chilled water was run through the core while the gel pre-ran at 300 V for 30 min. Samples were mixed with formamide in a 1:1 volume ratio, heated to 80°C for 5 min, and then plunged into an ice bath for 5 min before loading. The gel was run at 450 V for 4 h with chilled water running through the core. The gel was removed from the plates and stained in 1× SYBR Green II for 20 min. The gel was rinsed three times with ultrapure water and imaged in a GelDoc Go imager by Bio-Rad Laboratories. Desired bands were excised, crushed with a plastic dowel, and soaked in molecular grade water on a rotator for 24 h. The gel extract was filtered with a 0.22-µm syringe filter, concentrated in a 60°C vacuum centrifuge for 30 min, and desalted with NAP-5 columns according to the manufacturer’s instructions.

### Polymerase chain reaction

DNA was amplified using 1× reaction buffer (from various kits, the reaction buffer always was paired with the polymerase used), 200 µM dNTPs, 300 nM each forward and reverse primers, and 0.02–0.04 U/µl polymerase. If ET SSB was used, it was added to a concentration of 4 ng/µl. For amplifications with Phusion, DeepVent, and DeepVent (exo−), the T100 thermocycler from BioRad was set to perform an initial denaturation step at 95°C for 2 min, followed by 30 cycles of 95°C for 30 s, 58°C for 30 s, and 72°C for 15 s. A final extension at 72°C was performed for 5 min followed by a 4°C hold. For the amplifications with SD Polymerase, 3 mM MgCl_2_ was added and the thermocycler program set to perform an initial denaturation step at 92°C for 2 min, followed by 30 cycles of 92°C for 30 s, 58°C for 30 s, and 68°C for 15 s. A final extension at 68°C was performed for 5 min followed by a 4°C hold.

### Agarose gel electrophoresis

Polymerase chain reaction (PCR) products were run on a 2% agarose gel in 1× TBE. One hundred base pair ladder was loaded into the first well. The gel was run at 95°C for 45 min. Any desired bands were excised and purified with the PureLink Quick Gel Extraction Kit from Invitrogen.

### Sanger sequencing

Gel-purified PCR products were diluted to ≈2 ng/µl in molecular grade water. Twenty-five picomoles of either forward or reverse primer was added. Sequencing was performed with the “Difficult Template” Sanger sequencing service provided by Genewiz. Sequencing data were analysed using SnapGene.

### Oxford Nanopore sequencing

Gel-purified PCR products were prepared for sequencing using the Rapid Barcoding Kit V14 available from Oxford Nanopore Technologies according to the amplicon sequencing protocol provided by the manufacturer. The prepared libraries were sequenced using the MinION Mk1B device from Oxford Nanopore Technologies according to the manufacturer’s instructions. High-accuracy basecalling was performed with the Dorado basecaller in the MinKNOW software provided by Oxford Nanopore Technologies. Data analysis and variant calling were performed with the Amplicon workflow leveraging Medaka in the Epi2Me software available from Oxford Nanopore Technologies. The sampling size was reduced to 15 000 reads per barcode. Other parameters for the Oxford Nanopore sequencing analysis can be found in Supplementary Table S2.

## Results

### DNAzyme characterization

The E47 DNAzyme [23] was selected as the best candidate for ligating two DNA strands end-to-end after searching the DNAzyme database DNAmoreDB [24]. Several ligating DNAzymes exist, but the majority of the ones in the database form a branched DNA product [24–26]. In other cases, the ligating DNAzymes are long, such that synthesis becomes more arduous and expensive [23] or are highly sequence dependent [27]. The catalytic region of the E47 DNAzyme is only 24 nt long, with 10- and 13-nt-long binding arms for hybridizing the two subunit DNA strands together (Fig. 2A). A 5′ OH group is required on the S2 subunit, and a 3′ phosphate group is required on the S1 subunit. The 3′ phosphate is activated with imidazole and EDC to generate a reactive phosphorimidazolide. Then, the activated S1 unit is purified and mixed with the S2 subunit, the E47 catalytic strand, and Zn^2+^ as a cofactor. Only four nucleotides of the subunits to be ligated interact with the catalytic region of the E47 DNAzyme; it seemed reasonable to explore whether the binding arm sequences of the E47 DNAzyme could be changed as long as the four nucleotides in the catalytic region remained.

**Figure 3.**
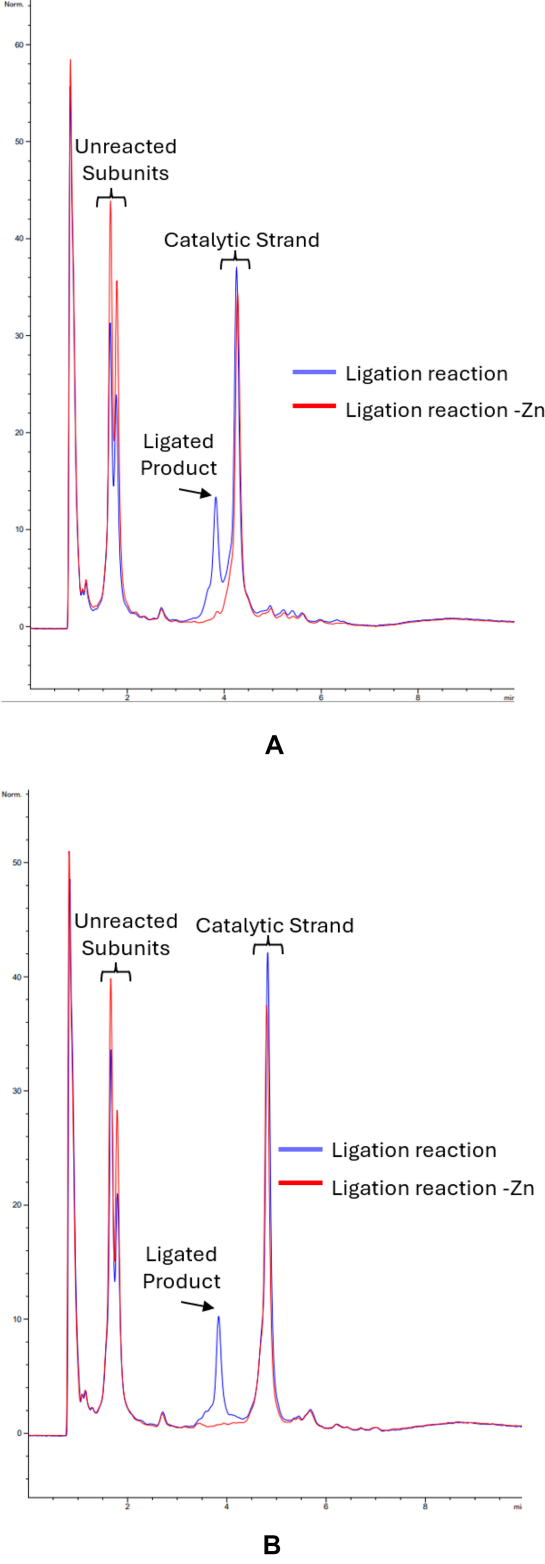
(**A**) HPLC chromatograms of ligation products with the original reported sequence. Blue line: Ligation reaction with all components. Red line: Ligation reaction without Zn cofactor. (**B**) HPLC chromatograms of ligation products with modified subunit sequences. Blue line: Ligation reaction with all components. Red line: Ligation reaction without Zn cofactor.

Sequence specificity of the E47 DNAzyme: The 5′ G on the S2 strand and the last three nucleotides on the 3′ end of the S1 strand seem to interact with the catalytic region of the DNAzyme. However, the other nucleotides in the substrate strands seem to be mostly involved in hybridization to the DNAzyme, and may not play a role in the catalysis. We explored the sequence specificity of the E47 DNAzyme by changing the binding arm sequence of the substrates and DNAzyme to random sequences (Fig. 2B). The resulting ligation products were analyzed with HPLC. The ligation product peaks were strong in both samples, indicating that the binding arm regions of the E47 DNAzyme can tolerate sequence modifications (Fig. 3A and B).

The sequence specificity of the catalytic 4-nt motif was also probed (Fig. 2C). Each nucleotide was either changed to its complement, maintaining the base pair identity in that position ( i.e. G → C or T → A), or switched to the other nucleotide that maintained the pyrimidine/purine identity in that position (i.e. C → T or G → A). Any of these modifications to the 4-nt catalytic motif resulted in a complete loss of ligation activity as measured with HPLC, with the exception of the 3′ T in the catalytic strand. Modifications of the 3′ T resulted in reduced catalytic activity and unexpected ligation products (Table 1 and Supplementary Fig. S5A–C). While the E47 DNAzyme can ligate a variety of sequences, the S1 strand must have the motif AGC at its 3′ end, while the S2 strand must have a G in the 5′ end position.

**Table 1.** Ligation activity of catalytic four-base motif mutations

Catalytic region sequence	Activity
(5′ → 3′)	
CGCT (original)	High (relative peak area 100%)
**G** GCT	None observed
**T** GCT	None observed
C**C**CT	None observed
C**A**CT	None observed
CG**G**T	None observed
CG**T**T	None observed
CGC**A**	Reduced (relative peak area 16%)
CGC**C**	Reduced, unexpected ligation products
	(relative peak area 50%)

#### Necessity of purification step between activation and ligation

There are two steps in performing ligation with this DNAzyme: activation of the 3′ phosphate group with imidazole and EDC, and ligation. Historically, a purification step was included between the activation and ligation steps to remove unreacted EDC and imidazole. The purification step is time consuming, sometimes leading to inactivation of the unstable phosphorimidazolide before the ligation could take place. The purification step also adds cost of purification materials such as columns, and results in the loss of some subunit due to column retention. We explored removing this step as well as the possibility of combining the activation and purification steps in a one-step reaction.

**Figure 4.**
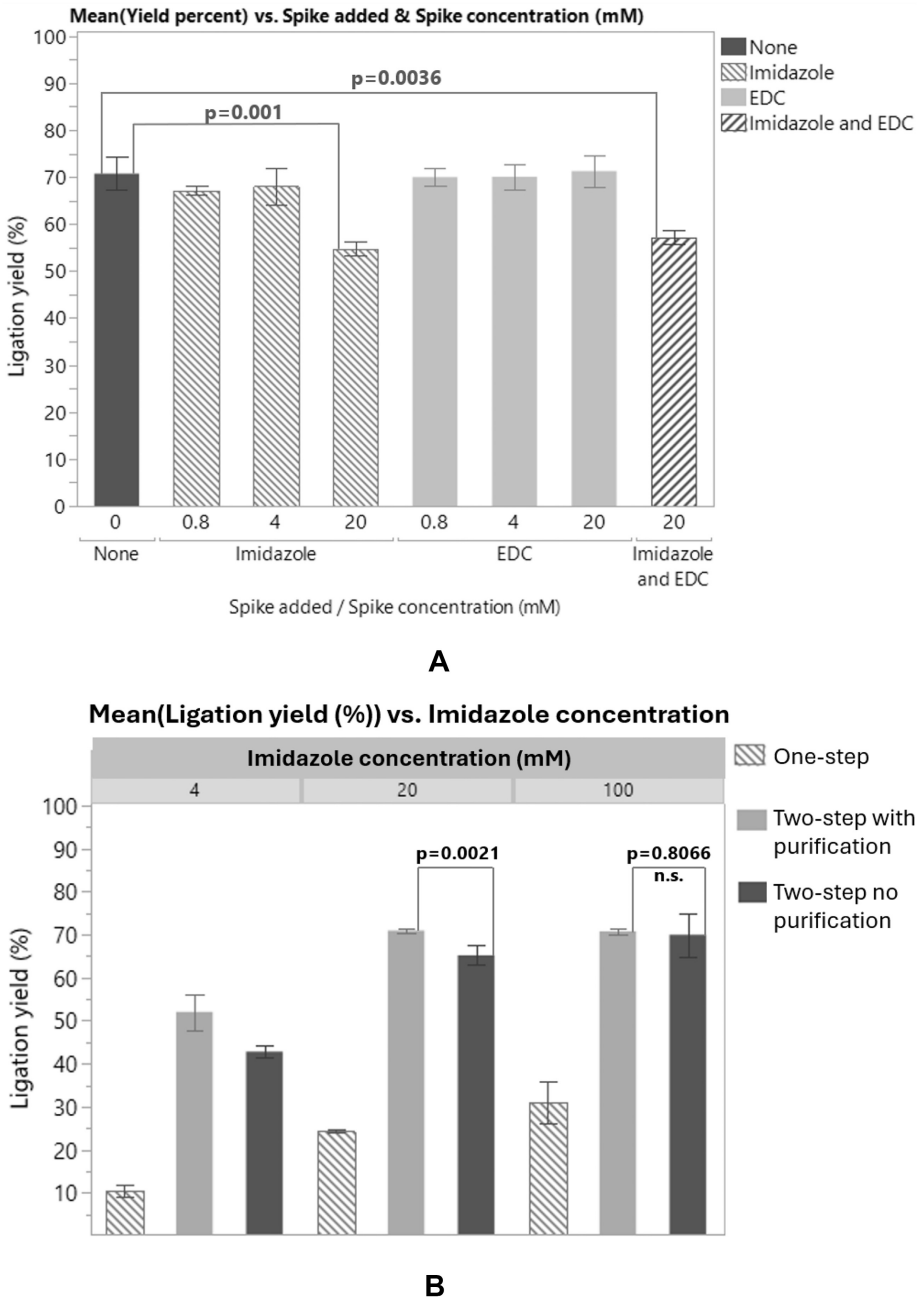
(**A**) Varying amounts of EDC, imidazole, or both EDC and imidazole were spiked into the ligation reaction. The final concentrations of EDC and imidazole after spiking the ligation solutions were plotted versus calculated yield as quantified by HPLC. (**B**) The purification step between activation and ligation steps was removed, and/or the activation and ligation steps were combined in a one-step reaction. Varying levels of imidazole were used to activate the S1 substrate. No significant difference was observed between column purified and non-purified samples if 100 mM imidazole was used for activation.

Purification between the activation and ligation steps is desired if one or both of the activation reactants (EDC or imidazole) inhibit the subsequent ligation reaction. To probe the inhibitory nature of EDC and imidazole, ligation reactions were spiked with EDC, imidazole, or EDC + imidazole. Results show that while spiking the ligation reaction with EDC it did not have a notable effect on the ligation reaction, imidazole showed a notable inhibitory effect (Fig. 4A). Next, ligation reactions were attempted without the use of a purification step between the activation and ligation steps, with varying amounts of imidazole in the activation step. Removal of the purification step did not significantly reduce the yield when 100 mM imidazole was used, and reducing the imidazole concentration by from 100 to 20 mM in the activation reaction still showed good activation of the substrate whether or not purification was used (Fig. 4B). Combining the activation and ligation steps resulted in a marked reduction in ligation activity (Fig. 4B and Supplementary Figs S1A and S2B), even with reduced imidazole concentrations. The activation and ligation reactions could therefore not practically be combined in a one-pot reaction, but if desired, the purification step between the activation and ligation steps can be removed with minimal or no impact to the yield.

#### Assessment of multi-piece assembly capabilities

A two-component system was designed to assess the capacity of the E47 DNAzyme to perform a multi-piece assembly. A substrate strand of 44 nucleotides was designed with a 3′ phosphate modification. A catalytic strand was designed to hybridize to the 3′ and 5′ ends of the substrate strand, such that the designed substrate would serve as both the S1 and S2 subunits. The number of the assembled strands was not limited by the sequence design; an indefinite number of substrates could be assembled. After a 3-h ligation period, the crude product was run on a denaturing PAGE gel with a single-stranded DNA ladder. The larges bands readily visible corresponded to an eight-piece assembly (Supplementary Fig. S4).

### First-tier ligation: attaching linkers to the symbols

**Figure 5.**
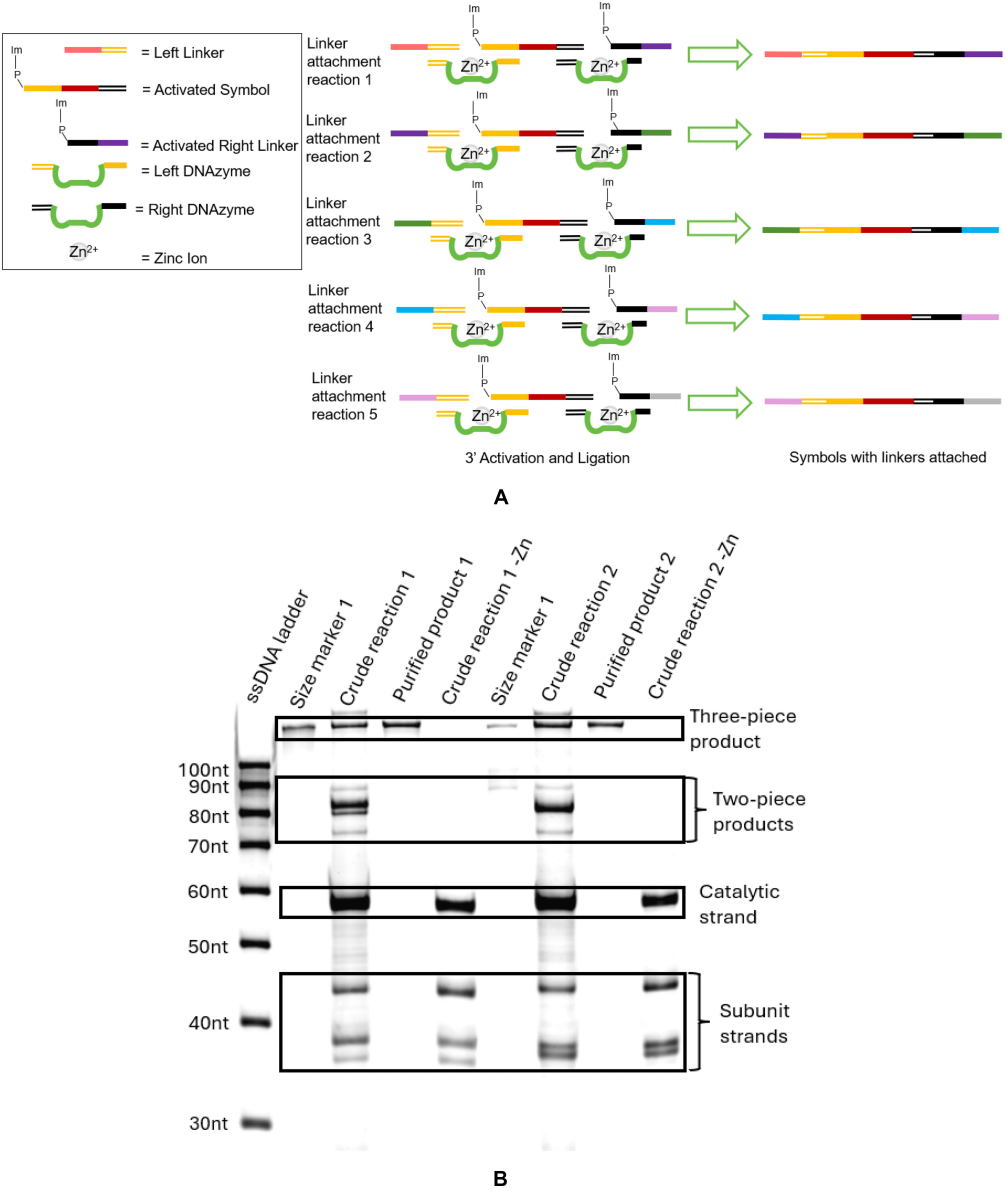
(**A**) Schematic of three-piece ligation and gel image of three-piece assembly. (**B**) Gel image of two different three-piece assemblies, including size markers, crude reaction, purified ligation product, and crude reaction with no Zn cofactor.

The first step in assembling the DNA data storage gene in our schema is to attach unique linkers to each symbol. Each symbol was one of three sequences, designated as symbols A, B, or C. The linkers attached to each symbol were selected based on the desired order of the symbols in the final assembly. For example, in order to have the symbols assembled in the order A → B → C → B → A, the symbols would have to be matched to the linkers that would attach them in that order (Table 2). Each linker possesses two regions: one region that connects to the symbol, and one that connects to another linker (Fig. 5A). The symbol-connecting region is the same for each linker, allowing any linker to be attached to any symbol. Conversely, the linker-connecting region is different for each, allowing only two specific linkers to be joined to each other. Sequences of each strand can be found in the Supplementary data. The linker–symbol assembly requires a three-piece ligation reaction involving three substrate strands and two unique DNAzymes (Fig. 5A). Analysis of the products with gel electrophoresis showed that the three-piece ligation was successful (Fig. 5B), and the full-length product was purified from the reactants using either HPLC or gel electrophoresis. The yield of the three-piece assemblies as quantified by HPLC was ∼40% ± 2% (Supplementary Fig. S2A and B). Five different linker–symbol complexes were prepared in this manner. At this stage PCR was not performed, because for the next ligation reaction the pieces need to have a 3′ phosphate. PCR amplification only results in 3′ OH groups. In addition, PCR results in double-stranded DNA, whereas for the next ligation reaction, ssDNA is required.

**Table 2.** The order of the data-encoding symbols was directed via the linker sequences

Symbol order in the final	Linkers added to each symbol in	Catalytic strands used to join
data storage gene 5′ → 3′	assembly step 1	the linkers in assembly step 2
Data gene 1:	Symbol A, left linker 1, right linker 1	Joins right linker 1 to left linker 2
A, B, C, B, A	Symbol B, left linker 2, right linker 2	Joins right linker 2 to left linker 3
	Symbol C, left linker 3, right linker 3	Joins right linker 3 to left linker 4
	Symbol B, left linker 4, right linker 4	Joins right linker 4 to left linker 5
	Symbol A, left linker 5, right linker 5	–
Data gene 2:	Symbol B, left linker 1, right linker 1	Joins right linker 1 to left linker 2
B, C, A, C, B	Symbol C, left linker 2, right linker 2	Joins right linker 2 to left linker 3
	Symbol A, left linker 3, right linker 3	Joins right linker 3 to left linker 4
	Symbol C, left linker 4, right linker 4	Joins right linker 4 to left linker 5
	Symbol B, left linker 5, right linker 5	–
Data gene 3:	Symbol B, left linker 1, right linker 1	Joins right linker 2 to left linker 1
C, B, C, A, B	Symbol C, left linker 2, right linker 2	Joins right linker 1 to left linker 4
	Symbol A, left linker 3, right linker 3	Joins right linker 4 to left linker 3
	Symbol C, left linker 4, right linker 4	Joins right linker 3 to left linker 5
	Symbol B, left linker 5, right linker 5	–

The order was changed by either changing the symbol–linker pairings in the first assembly step as for storage gene 2, or by changing the catalytic strand sequences in the second assembly step as for storage gene 3.

### Second-tier ligation: assembling the data symbols

#### Five-piece DNA assembly

**Figure 6.**
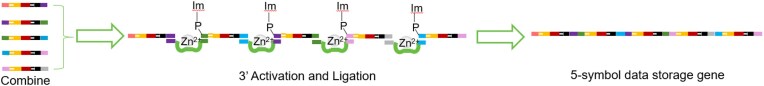
Five symbols with linkers attached were assembled in a final ligation reaction to create a five-symbol data storage gene.

The five symbol–linker complexes were assembled in a second ligation reaction, with four DNAzymes designed to hybridize to the linker ends and join them in a specified order (Fig. 6), resulting in a five-symbol data storage gene. The results were verified by gel electrophoresis and the full-length product purified from the gel (Supplementary Fig. S6). The yield for the five-symbol assemblies was ∼0.082% ± 0.008% as quantified by PAGE (Supplementary Fig. S3A and B). The five-piece product was amplified by PCR and the product purified via an agarose gel.

#### PCR optimization of the assembled DNA data storage gene

**Figure 7.**
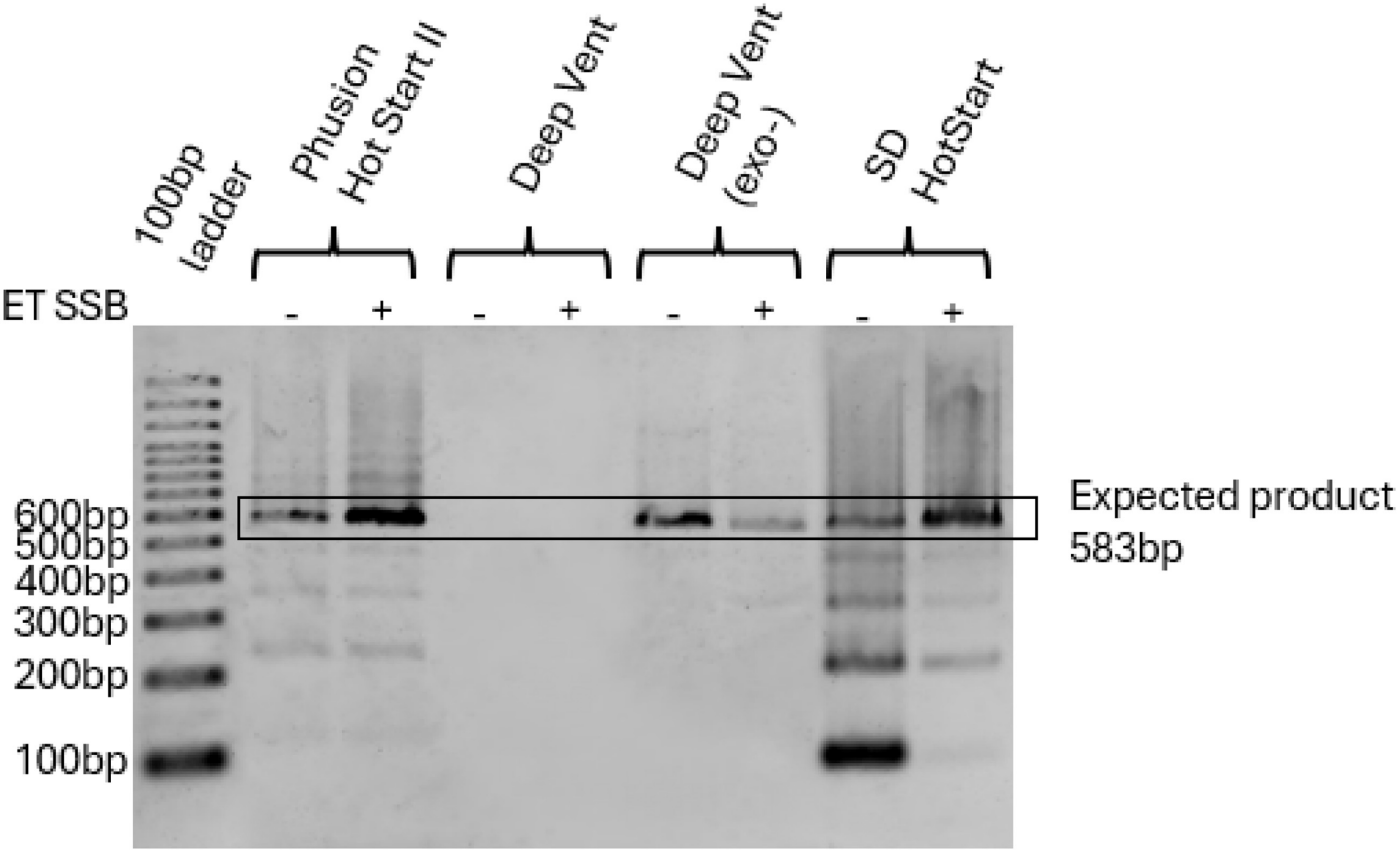
PCR of a five-symbol data storage gene assembly with different polymerases with and without ET SSB. Expected gene length is 583 bp.

PCR of the five-piece assembly with Phusion polymerase results in a ladder banding pattern due to the repeats of the linker attachment sequence in the template (Fig. 7) [28]. Changing to a different DNA polymerase with strand displacement activity may result in a reduction of the ladder banding [28]. Several different DNA polymerases were probed, in addition to a single-stranded DNA binding protein (ET SSB) that is known to reduce secondary structure formation in difficult PCR templates [28]. ET SSB addition had positive impacts on amplification with Phusion and SD HotStart, but a negative impact on amplification with DeepVent (exo−). Phusion polymerase and SD Polymerase showed strong amplification but lots of laddering (Fig. 7). DeepVent polymerase did not show any amplification, but a DeepVent polymerase modified to remove the 3′ → 5′ exonuclease showed good amplification with marked reduction in laddering (Fig. 7 and Supplementary Fig. S7). Three unique assemblies were prepared and amplified with DeepVent exo– polymerase, resulting in strong amplification of the full-length assembly and minimal laddering (Supplementary Fig. S7).

#### Order of the data storage symbols directed via the linker ends

**Figure 8.**
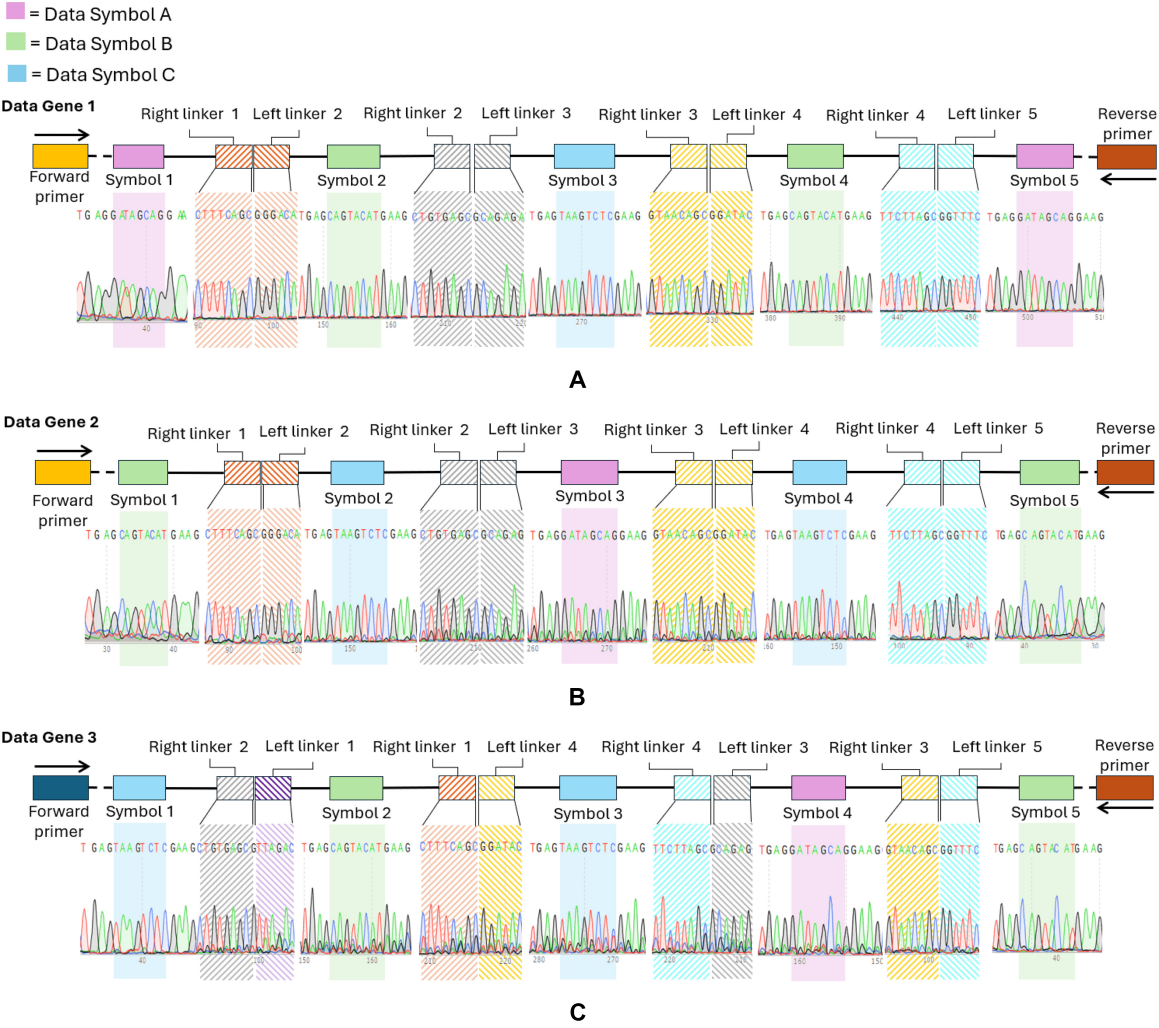
(**A**) Sequencing data with symbol and transition areas highlighted. (A) Data gene 1. (**B**) Data gene 2 with symbols arranged in a different order via the linker sequences. (**C**) Data gene 3 with symbol + linker pieces arranged in a different order via the DNAzyme sequences.

The order of the data-encoding DNA symbols can be directed by changing the linker ends attached to each symbol. Two data storage genes were prepared, and the order of the symbols was directed by switching the symbol–linker pairing in the first-tier assembly (Table 2). The symbol–linker complexes were assembled in a second-tier assembly reaction, and the resulting full-length product was purified and amplified as before. The two five-symbol data storage genes were sequenced and the correct symbol order verified (Fig. 8A and B).

A third five-symbol data storage gene was assembled, but this time the order of the symbols was shuffled by changing the sequences of the DNAzymes used in the second assembly step. The symbol–linker complexes used for this gene were the same as for the second gene, but the DNAzymes were designed to link them in a different order (Table 2). The Sanger sequencing data in Fig. 8C show that the third data gene was successfully assembled by changing the sequences of the DNAzyme catalytic strands in the second assembly step.

While the Sanger sequencing data showed the expected sequence, secondary signals were visible throughout the traces. We suspected this was due to the artifacts caused by the amplification of templates containing repeats, as was visible in Fig. 7. To investigate further, Oxford Nanopore sequencing was also performed. No variants were detected in any of the assemblies, which had an average coverage of around ∼11 000× for each sample (Supplementary Fig. S8A–D). These results confirmed that each of the three storage genes had been assembled as desired using the catalytic splint ligation method.

## Discussion

We have demonstrated a novel DNA assembly method that can be leveraged for DNA data storage. The protein-free catalytic splint assembly approach reduces the cost of the assembly reagents, and the symbol-linker approach enables the use of bulk DNA oligos, which reduces the cost associated with synthesizing data storage oligos on demand [18].

This chemical ligation method has other unique advantages; for example, unlike other chemical ligation methods such as click chemistry, which introduce unnatural linkages in the DNA backbone, DNAzyme ligation preserves the natural phosphodiester backbone of DNA [29]. The stability of the triazole linkages formed during click ligation during long-term storage has not yet been explored, nor has their ability to be acted on by restriction endonucleases or the CRISPR/Cas editing system, which may be used in compute or editing operations. In addition, the catalytic splint ligation method only requires one oligo to have a modification (3′ phosphate), while click ligation requires modifications on both oligos (azide on one oligo, alkyne on the other) [30]. Having fewer chemical modifications results in cheaper DNA, which is crucial for data-storage-scale DNA assembly. The cost of the oligos required for catalytic splint ligation is very similar to the oligos required for traditional splint ligation: Both methods require two substrate strands and a splint strand, and the modifications required both entail a phosphate on one substrate strand and an –OH on the other substrate strand. Using a catalytic splint removes the need for a ligase enzyme, drastically reducing the cost of the assembly.

The encoding density can be improved by reducing the length of the binding arms of the linkers and symbols. Previous work has shown successful ligation with the E47 DNAzyme with only 9-nt binding arms [31], little more than half of what we used in this study, and work with cleavage DNAzymes has shown successful activity with only 7-nt binding arms [32]. Reducing the binding arm length can drastically increase the density of the method we demonstrate in this work, provided the yield of the assembly steps is not compromised. Since DNA is already so dense, we believe the trade-offs between encoding density and reduced cost/improved write speed in our proposed system are acceptable.

DNA data storage will mostly be used for archival applications, where reading will be infrequent. When the data are read, any sequencing method may be used; Oxford Nanopore is a particularly attractive option since it has a broad read length range [33] (https://nanoporetech.com/document/requirements/lig-seq-input-load). Oxford Nanopore has been shown to be suitable for DNA data storage applications, particularly with the use of error correcting codes similar to those used in traditional data storage [34–37]. Sequencing methods like Oxford Nanopore are continually evolving; the accuracies, costs, and throughput will continue to improve over time.

Future work will be needed to increase both the yield and the rate of the assembly steps. The five-piece assemblies had a lower-than-expected yield compared to those of the two- and three-piece assemblies. As the assembled DNA constructs grow longer and have more pieces, a longer reaction time or increased reaction rate would likely be needed in order to maintain yields on par with shorter assemblies with fewer pieces. Longer DNA molecules will tend to form secondary structures, and each sequence will have a harder time finding its complement in a complex mixture of many sequences. Increasing the catalytic rate of the DNAzyme will be key to improving the reaction yield over the course of a reasonable reaction time. Past studies have shown that DNAzyme performance can be drastically improved through the optimization of the DNAzyme sequence and reaction conditions; take, for example, the I-R1 DNA-hydrolyzing DNAzyme [38], whose *k*_obs_ was improved 10× by two single-base mutations in the catalytic core, or the 10-23 DNAzyme whose activity under physiological conditions was improved 20× through the introduction of modified nucleotides into its core [32]. The catalytic efficiency of the metallizing DNAzyme PS5.ST1 was improved over 400× through sequence and reaction condition optimizations [39, 40]. In addition, exploration of novel DNA ligating DNAzymes could also result in new DNAzymes that have a faster activity or higher yield than E47. Improving the ligating DNAzyme yield and rate can enable further assembly tiers to attain the geometric growth of the storage gene as proposed in the “Introduction” section, and can also enable the assembly of more pieces in a single reaction. Even if the final assemblies have a low yield, a PCR reaction is desirable in any case in order to both amplify the DNA and render it to a double-stranded state, which is more stable for long-term storage [41].

Automation of the process we describe here will be necessary for assemblies at data storage scales. The assembly method in our work requires mostly mixing steps, which are simple to automate. The activation and ligation reactions are performed at room temperature, and as we demonstrated in Fig. 3, the column purification step between activation and ligation steps can be removed. One exception that would be necessary to explore in future work is to simplify the purification step between the first assembly and subsequent assemblies. In this work, we used PAGE or HPLC purification to remove leftover reactants and catalytic strands, which would not be suitable for large-scale automation. Magnetic bead-based purification strategies are an attractive option, and these are commonly automated with liquid-handling robots for NGS library prep and other large-scale operations [42, 43]. In addition to liquid-handling robots, lab-on-a-chip platforms are also an attractive option for automation and scale-up of the write process [44–46]. It would also be desirable to stabilize the activated strands, so that they can be stored in the active state rather than having to be activated immediately prior to assembly. The design of the DNA sequences to populate larger linker, symbol, and DNAzyme libraries can also be automated [47]: once desired pools of linkers, symbols, and DNAzymes are designed, they can be synthesized and stored in bulk.

Catalytic splint ligation using E47 could potentially be used for gene assembly for genomic applications; however, the necessity of the four-base motif that interacts with the catalytic region of the splint does limit its versatility. The required four-base motif does not pose a problem for data storage applications, since the required four-base motif is outside the encoding regions of the DNA strand, and sequences for DNA data storage are highly adaptable to the user’s needs. Future work could explore novel ligating DNAzymes that do not possess such stringent sequence dependence in the catalytic region. In addition, exploration of novel ligating DNAzymes could also result in new DNAzymes that are not inhibited by imidazole, or that have a faster activity or higher yield than E47.

Catalytic splint ligation is a promising avenue for DNA assembly, and particularly enables lower cost DNA data storage. Utilizing this assembly chemistry paired with the joining of prefabricated data encoding symbols via linker ends makes fast, lower cost DNA data writing more feasible.

## Supplementary Material

gkaf582_Supplemental_File

## Data Availability

The data underlying this article are available in the article and in its online supplementary material.
