# Supplementary material for: Directed assembly of single-stranded DNA fragments for data storage via protein-free catalytic splint ligation
Source: Nucleic Acids Res. 2025 Jun 30;53(12):gkaf582. doi: 10.1093/nar/gkaf582 (PMC12207399; doi:10.1093/nar/gkaf582)
Supplement: gkaf582_Supplemental_File [file gkaf582_supplemental_file.pdf]

SUPPLEMENTARY INFORMATION

S1A.

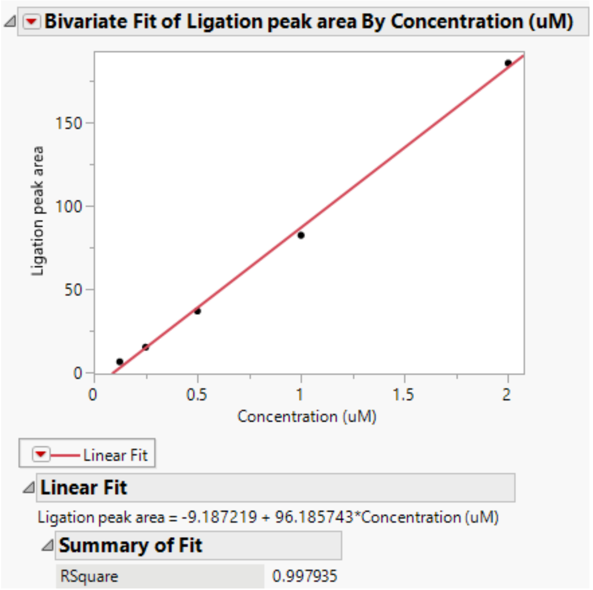

S1B.

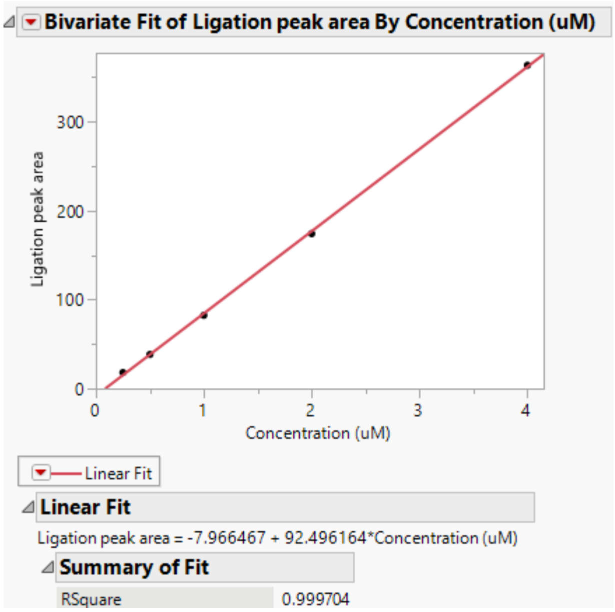

Supplementary Figure S1: Standard curves for HPLC quantification of two-piece ligation reaction yield. **S1A**: Standard curve for main text figure 4A. **S1B:** Standard curve for main text figure 4B.

S2A.

Bivariate Fit of Ligation peak area By Concentration (uM)

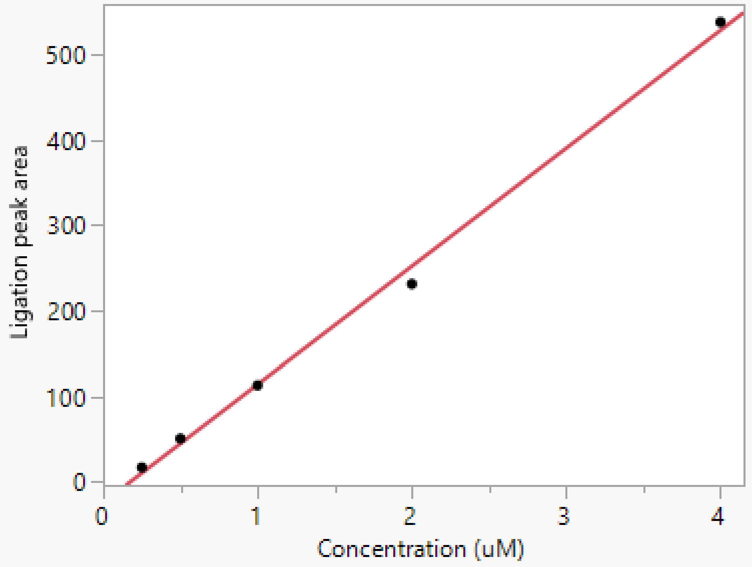

Linear Fit

Ligation peak area = -24.43303 + 138.22824*Concentration (uM)

Summary of Fit

RSquare	0.996603

Sample	Ligation peak time	Ligation peak area	Calc uM Ligation	Theoreticalyield (%) ligation
Tr1.5b_1	4.73	181.57	1.49	**40.94**
Tr1.5b_2	4.725	189.03	1.54	**42.42**
Tr1.5b_3	4.721	185.57	1.52	**41.74**

S2B.

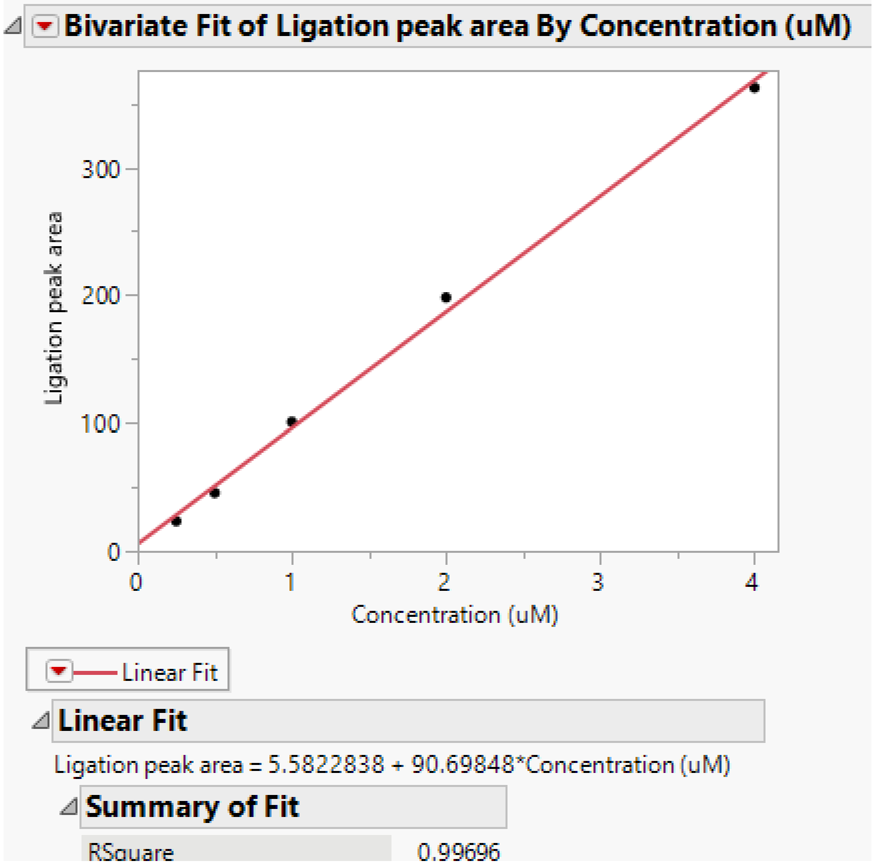

Sample	Ligation peak time	Ligation peak area	Calc uM Ligation	Theoretical yield (%) ligation
Tr1.1c_1	4.514	124.12	1.43	**39.29**
Tr1.1c_2	4.486	121.25	1.40	**38.42**
Tr1.1c_3	4.494	122.68	1.41	**38.85**

Supplementary Figure S2: Standard curves and HPLC quantification of three-piece assembly reaction yield. **S2A**: Standard curve and assembly yields for the quantification of three-piece assembly 1. **S2B:** Standard curve and assembly yields for the quantification of three-piece assembly 2.

S3A.

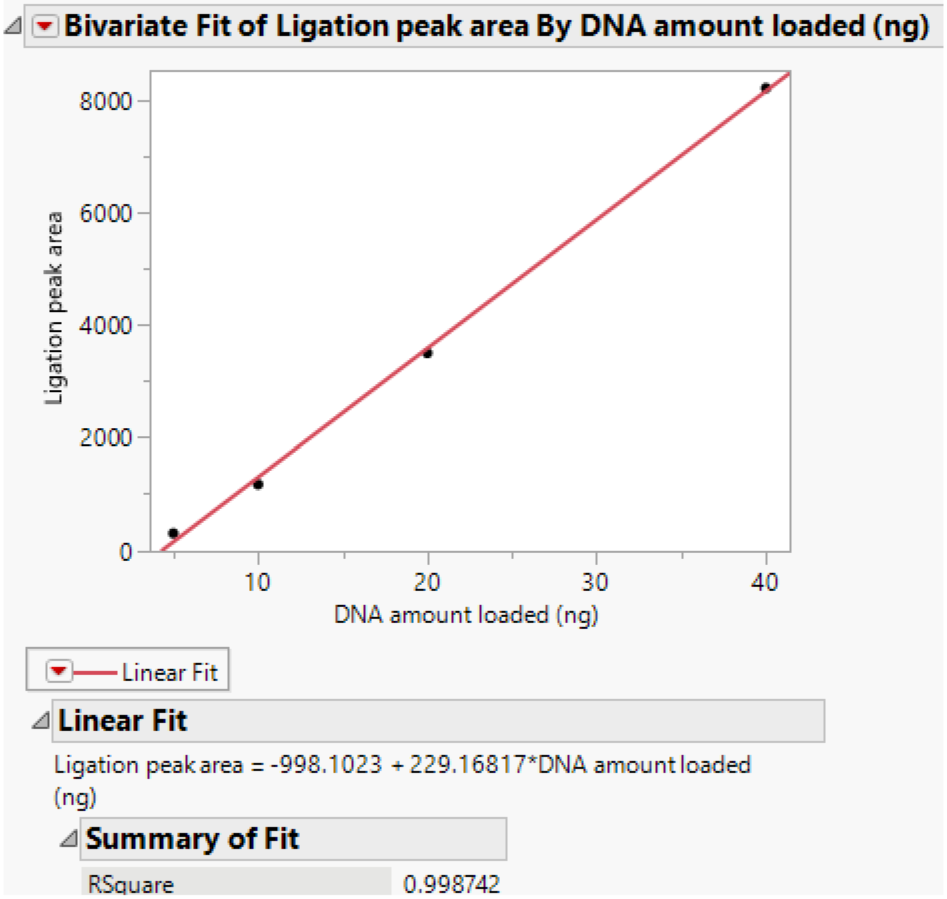

Sample	Area	ng	ng/uL	uM	Max yield (uM)	Yield %
5P_1	1263.426	9.868	0.493	0.003	3.636	**0.075**
5P_2	1422.376	10.562	0.528	0.003	3.636	**0.080**
5P_3	1750.740	11.995	0.600	0.003	3.636	**0.091**

S3B.

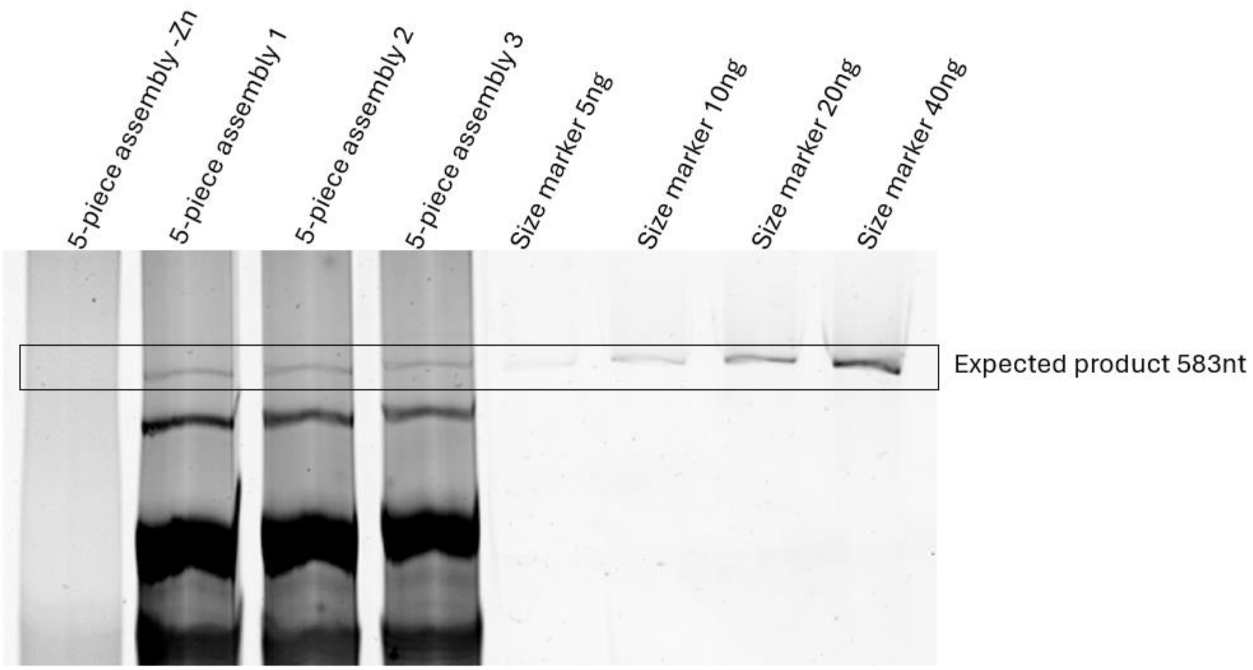

Supplementary Figure S3: Standard curve and PAGE quantification of five-piece assembly reaction yield. **S3A**: Standard curve and assembly yields for the quantification of five-piece assemblies. **S3B:** PAGE gel with the five-piece assembly crude reactions

S4.

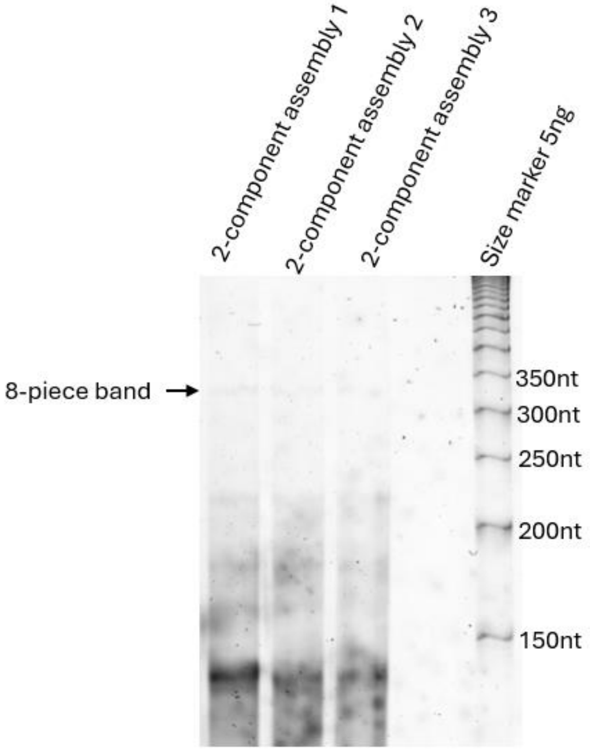

Supplementary Figure S4: A two-component system was used to see how many 44nt fragments could be assembled in a 3-hour ligation period. The largest visible band in the 12% PAGE gel was closest to the expected band for an 8-piece assembly.

S5A.

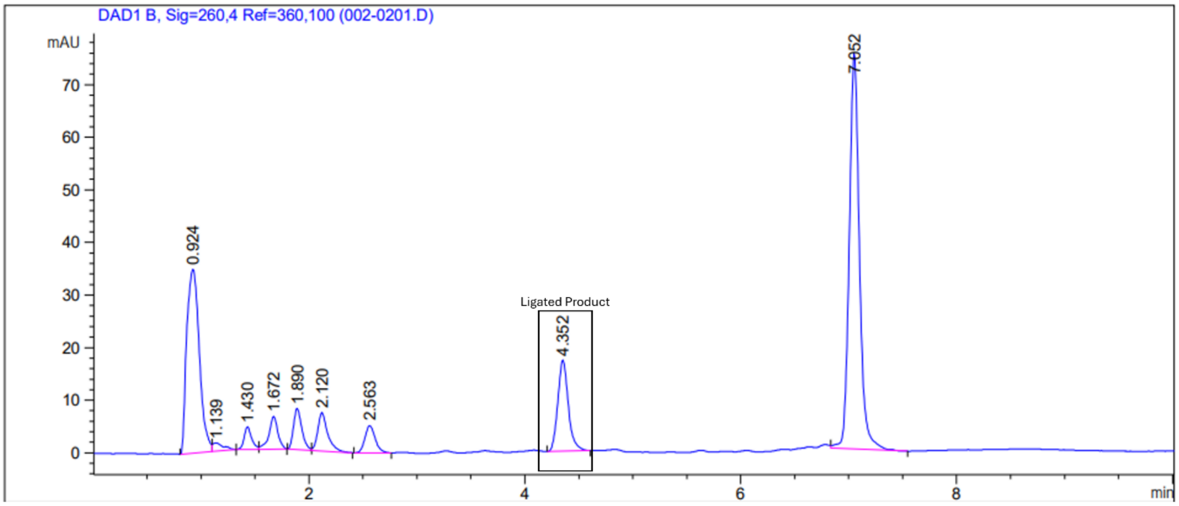

DAD1 B, Sig=260,4 Ref=360,100 (002-0201.D)
mAU
70
60
50
40
30
20
10
0
0.924
1.139
1.430
1.672
1.890
2.120
2.563
Ligated Product
4.352
7.052
2
4
6
8
min

S5B.

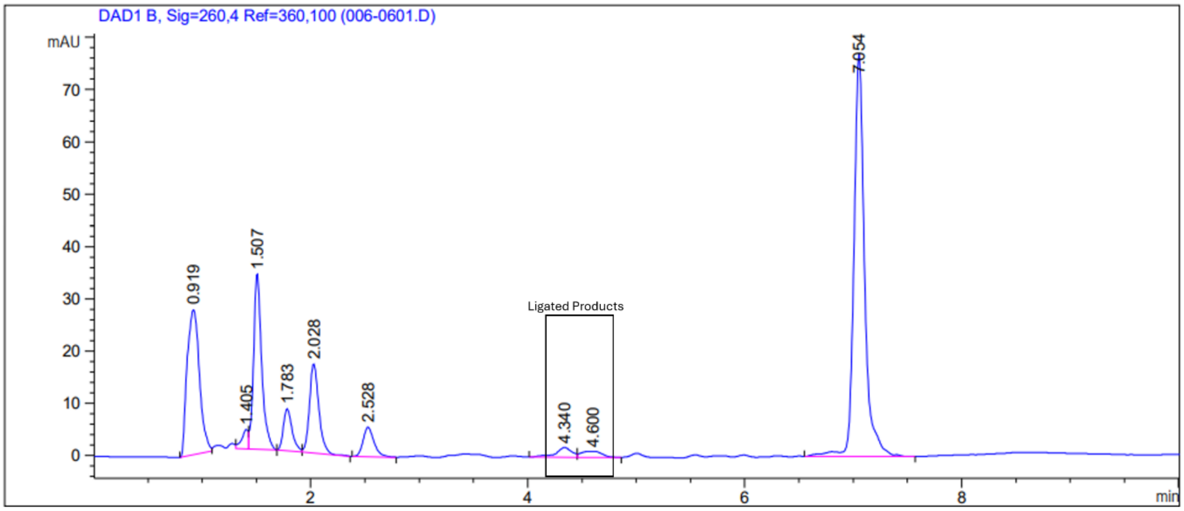

DAD1 B, Sig=260,4 Ref=360,100 (006-0601.D)
mAU
70
60
50
40
30
20
10
0
0.919
1.405
1.507
1.783
2.028
2.528
Ligated Products
4.340
4.600
7.054
2
4
6
8
min

S5C.

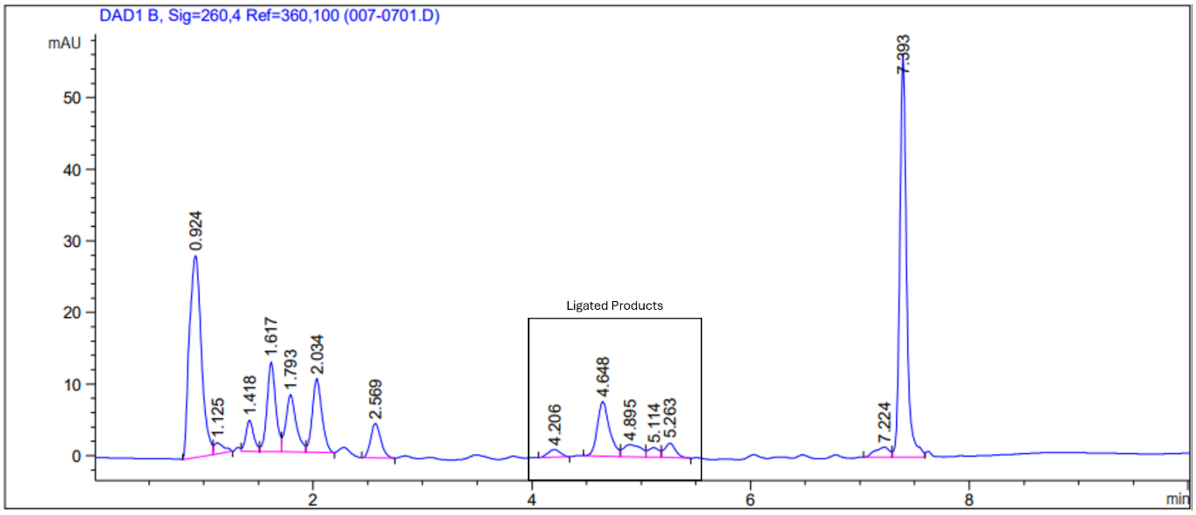

Supplementary Figure S5: HPLC Chromatograms of attempted ligations with mutated catalytic 4-base motif. **S5A**: Original E47 DNAzyme. **S5B**: 3′ T modified to an A. **S5C**: 3′ T modified to a C.

S6

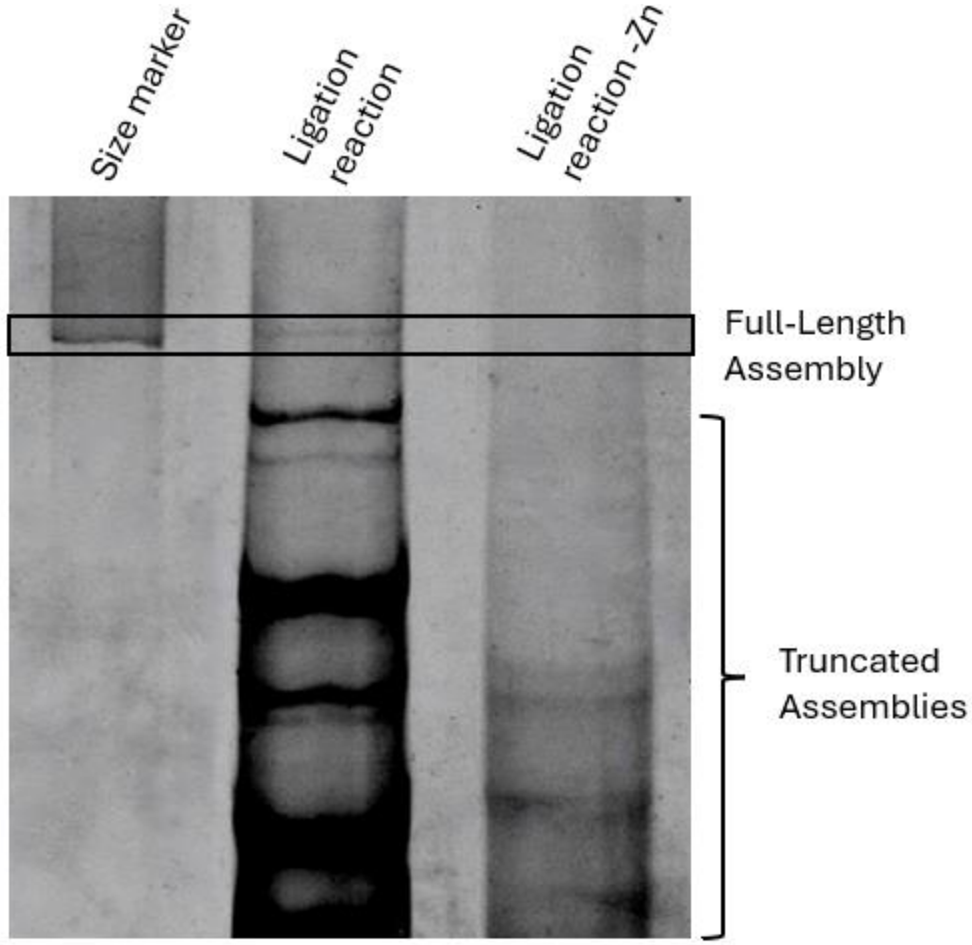

Supplementary Figure S6: Final assembly of five sub-assemblies. Lane 1: Size marker. Lane 2: Ligation reaction. Lane 3: Ligation reaction with no Zn cofactor.

S7.

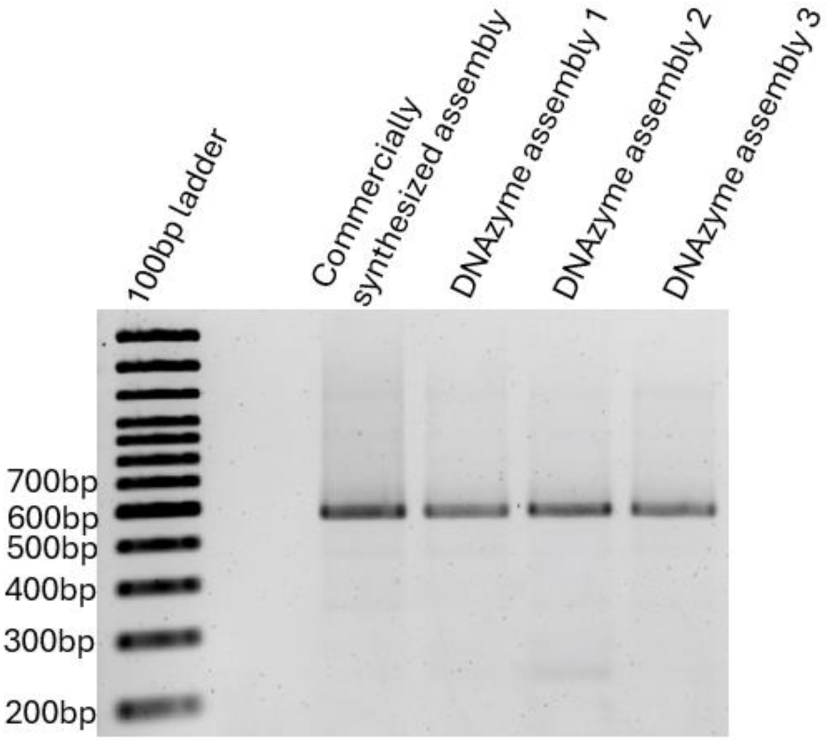

Supplementary Figure S7: PCR amplification of three data storage genes assembled with DNAzymes with DeepVent (exo-) polymerase.

S8A

Sample alias	Barcode	Reads	Bases	Median read length	Amplicons	Unmapped	Variants (indels)
**Assembly_1**	barcode01	15.0 k (33%)	7.5 M (33%)	500	1	1 (0%)	0 (0)
**Assembly_2**	barcode02	15.0 k (33%)	7.5 M (33%)	504	1	57 (0%)	0 (0)
**Assembly_3**	barcode03	15.0 k (33%)	7.4 M (33%)	492	1	4 (0%)	0 (0)

Supplementary Figure S8A: Summary of Oxford Nanopore sequencing results for all three assemblies

S8B

Assembly_1

Reads<br>15,000	Bases<br>7.50093e+06	Mean length<br>500.1
Mean quality<br>14.8	Amplicons detected<br>1 / 1	Mean coverage across all amplicons<br>11332.1
Smallest mean coverage for any amplicon<br>11332.1	SNVs<br>0	Indels<br>0

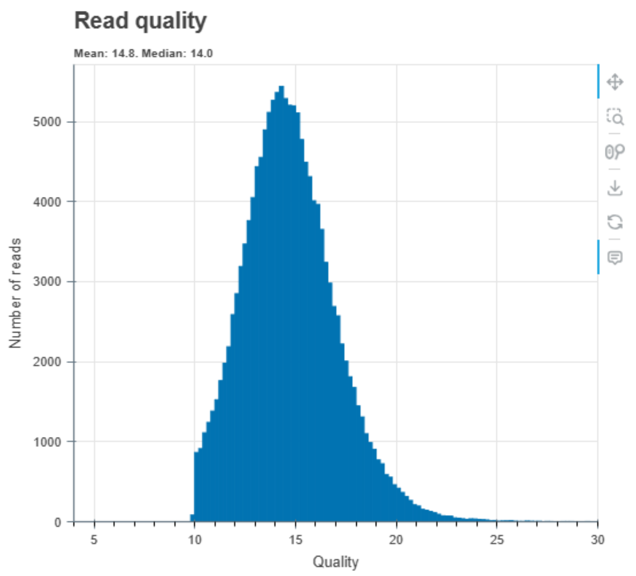

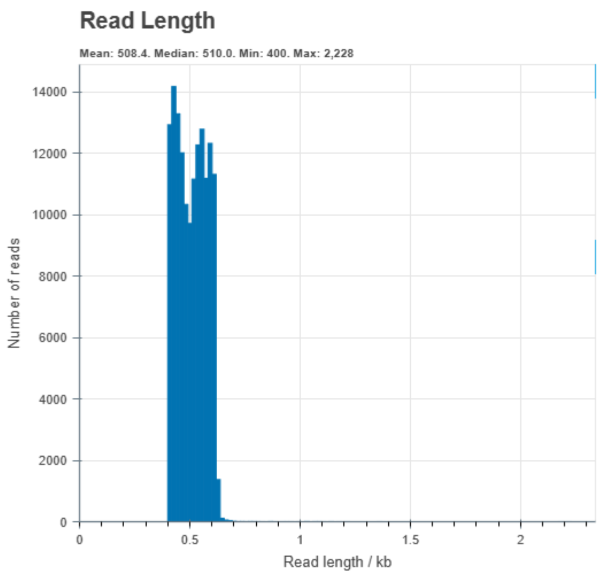

Depth of coverage

Coverage along the individual amplicon, (use the dropdown menu to view the plots for the individual amplicons).

Assembly_1_

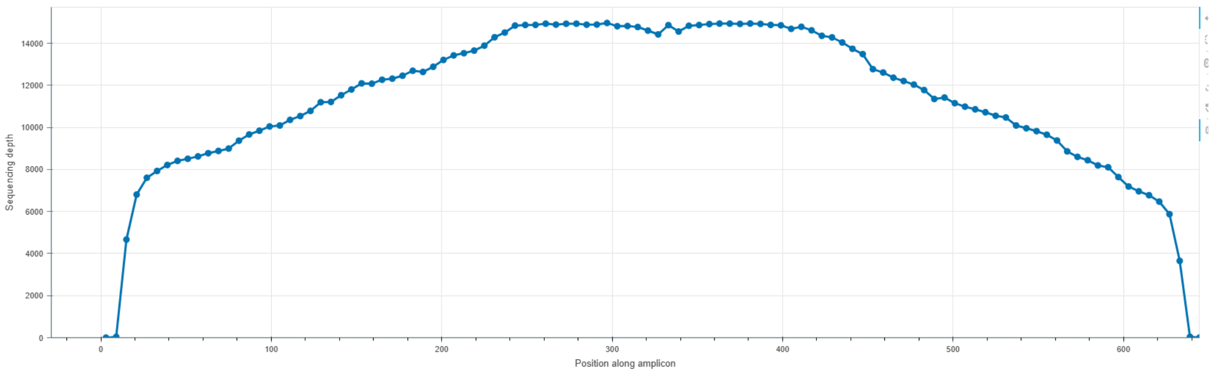

Supplementary Figure S8B: Key results and metrics for Assembly 1 Oxford Nanopore sequencing

S8C

Assembly_2 ▾

Reads	Bases	Mean length
14,998	7.51194e+06	500.9
**Mean quality**	**Amplicons detected**	**Mean coverage across all amplicons**
14.5	1 / 1	11279.7
**Smallest mean coverage for any amplicon**	**SNVs**	**Indels**
11279.7	0	0

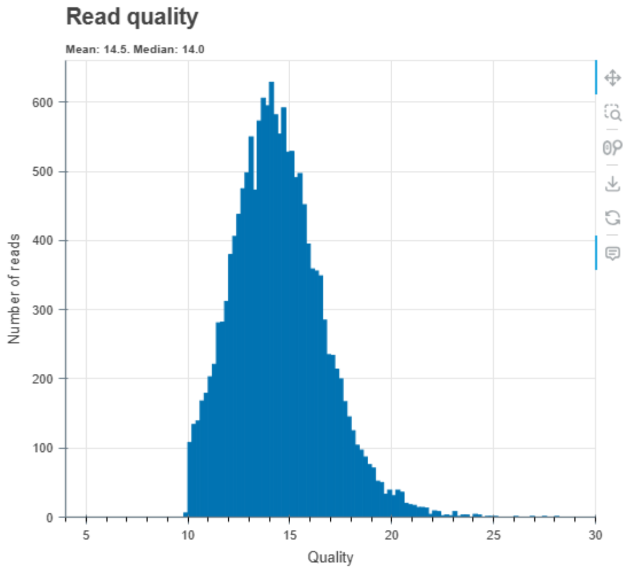

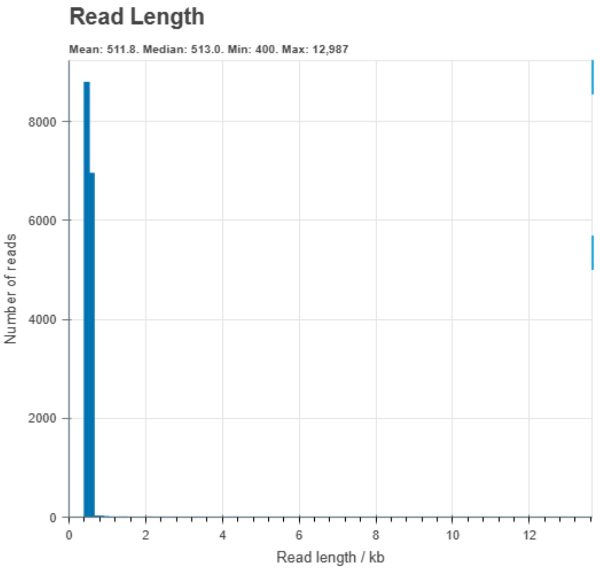

Depth of coverage

Coverage along the individual amplicon, (use the dropdown menu to view the plots for the individual amplicons).

Assembly_2_ ▾

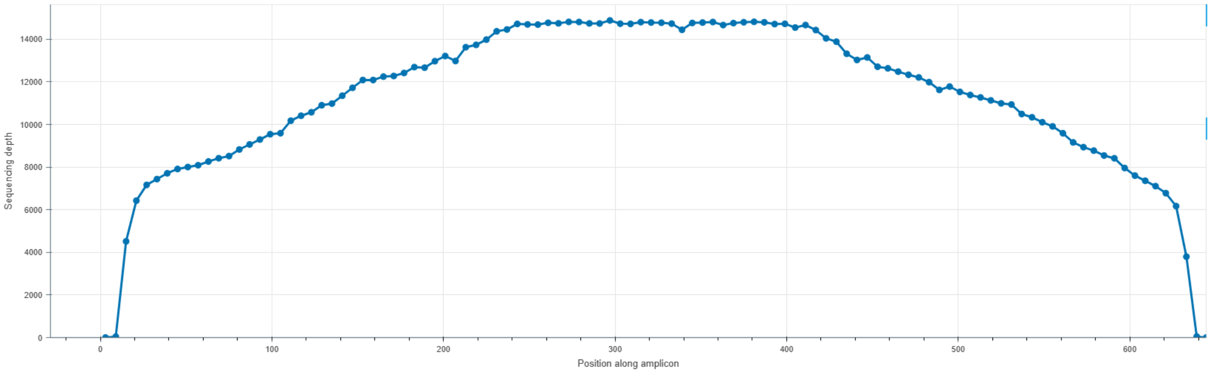

Supplementary Figure S8C: Key results and metrics for Assembly 2 Oxford Nanopore sequencing

S8D

Assembly_3

Reads	Bases	Mean length
14,998	7.41411e+06	494.3
**Mean quality**	**Amplicons detected**	**Mean coverage across all amplicons**
14.5	1 / 1	11100.1
**Smallest mean coverage for any amplicon**	**SNVs**	**Indels**
11100.1	0	0

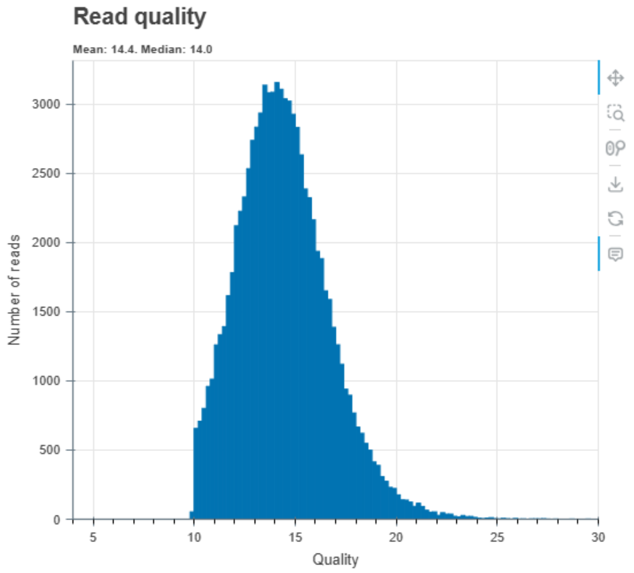

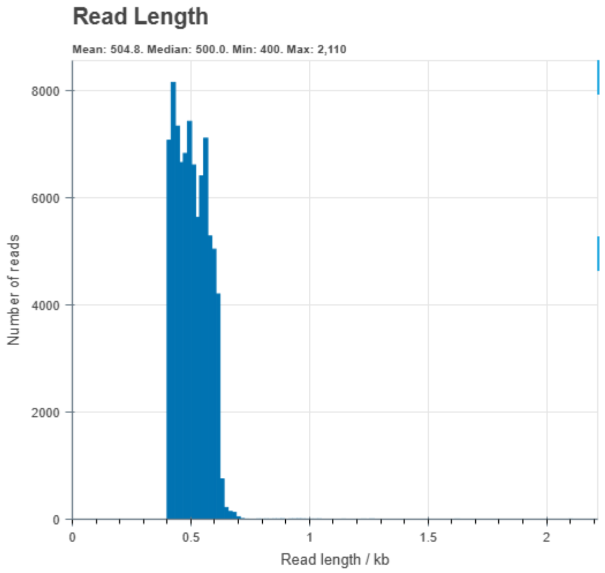

Depth of coverage

Coverage along the individual amplicon, (use the dropdown menu to view the plots for the individual amplicons).

Assembly_3_

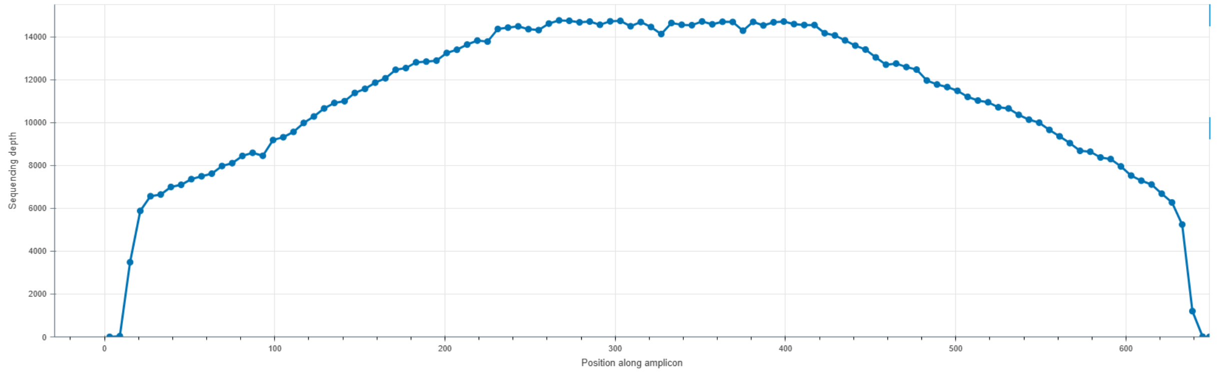

Supplementary Figure S8D: Key results and metrics for Assembly 2 Oxford Nanopore sequencing

Sequence name	Sequence (5'-3')
E47 Original Catalyst	CGGATAGTGTTCTTTCGCTAGACCATGTGACGCATGGTGAGATGCTT
E47 original S2	GGAACACTATCCG
E47 original S1 P	AAGCATCTCAAGC/3Phos/
E47 Catalyst C1modG	CGGATAGTGTTCTTTGGCTAGACCATGTGACGCATGGTGAGATGCTT
E47 S2 C1modG	CGAACACTATCCG
E47 Catalyst C1modT	CGGATAGTGTTCTTTTGCTAGACCATGTGACGCATGGTGAGATGCTT
E47 S2 C1modT	AGAACACTATCCG
E47 Catalyst G2modC	CGGATAGTGTTCTTTCCCTAGACCATGTGACGCATGGTGAGATGCTT
E47 S1P G2modC	AAGCATCTCAAGG/3Phos/
E47 Catalyst G2modA	CGGATAGTGTTCTTTCACTAGACCATGTGACGCATGGTGAGATGCTT
E47 S1P G2modA	AAGCATCTCAAGT/3Phos/
E47 Catalyst C3modG	CGGATAGTGTTCTTTCGGTAGACCATGTGACGCATGGTGAGATGCTT
E47 S1P C3modG	AAGCATCTCAACC/3Phos/
E47 Catalyst C3modT	CGGATAGTGTTCTTTCGTTAGACCATGTGACGCATGGTGAGATGCTT
E47 S1P C3modT	AAGCATCTCAAAC/3Phos/
E47 Catalyst T4modA	CGGATAGTGTTCTTTCGCAAGACCATGTGACGCATGGTGAGATGCTT
E47 S1P T4modA	AAGCATCTCATGC/3Phos/
E47 Catalyst T4modC	CGGATAGTGTTCTTTCGCCAGACCATGTGACGCATGGTGAGATGCTT
E47 S1P T4modC	AAGCATCTCAGGC/3Phos/
Symbol A	GGTAGGAGTTCACTGAGGATAGCAGGAAGCCTAGTATCTCAAGC/3Phos/
Symbol B	GGTAGGAGTTCACTGAGCAGTACATGAAGCCTAGTATCTCAAGC/3Phos/
Symbol C	GGTAGGAGTTCACTGAGTAAGTCTCGAAGCCTAGTATCTCAAGC/3Phos/

Primer binding site forward	GGTAGACAAGTGACCATTGACATTCTGAGTCCAGC/3Phos/
Right linker 1	GGAACACTATCTGTATGGATCTAAGAGTCTTTCAGC/3Phos/
Left linker 2	GGATACAATGATAGAGGTTGACATTCTGAGTCCAGC/3Phos/
Right linker 2	GGTTTCGTGGTAGTCATTTGACATTCTGAGTCCAGC/3Phos/
Left linker 3	GGAACACTATCTGTATGATTTATCCGTTCTGTGAGC/3Phos/
Right linker 3	GGGACAGTTCTCAATCTTTGACATTCTGAGTCCAGC/3Phos/
Left linker 4	GGAACACTATCTGTATGTGAGGATTCACGTAACAGC/3Phos/
Right linker 4	GCAGAGATTATGTGTAGTTGACATTCTGAGTCCAGC/3Phos/
Left linker 5	GGAACACTATCTGTATGTGATTGCCCTCTTCTTAGC/3Phos/
Primer binding site reverse	GGAACACTATCTGTATGCCTGATAACGATCAAGTTGT
Left linker attachment DNAzyme	CATACAGATAGTGTTCTTTCGCTAGACCATGTGACGCATGGTGAGATACTAG GCTTC
Right linker attachment DNAzyme	CTCAGTGAACTCCTACTTTCGCTAGACCATGTGACGCATGGGGACTCAGAA TGTCAA
Linker 1 to 2 DNAzyme	AGATTGAGAACTGTCCTTTCGCTAGACCATGTGACGCATGGGAAAGACTCT TAGATC
Linker 2 to 3 DNAzyme	CTACACATAATCTCTGTTTCGCTAGACCATGTGACGCATGGCACAGAACGG ATAAAT
Linker 3 to 4 DNAzyme	CCTCTATCATTGTATCTTTCGCTAGACCATGTGACGCATGGGTTACGTGAAT CCTCA
Linker 4 to 5 DNAzyme	ATGACTACCACGAAACTTTCGCTAGACCATGTGACGCATGGAAGAAGAGG GCAATCA
Linker 2 to 1 DNAzyme	CGATGGTCACTTGTCTAATTTCGCTAGACCATGTGACGCATGGCACAGAAC GGATAAAT
Linker 1 to 4 DNAzyme	CCTCTATCATTGTATCTTTCGCTAGACCATGTGACGCATGGGAAAGACTCTT AGATC
Linker 4 to 3 DNAzyme	CTACACATAATCTCTGTTTCGCTAGACCATGTGACGCATGGAAGAAGAGGG CAATCA
Linker 3 to 5 DNAzyme	ATGACTACCACGAAACTTTCGCTAGACCATGTGACGCATGGGTTACGTGAA TCCTCA
3-piece size marker 1	GGTAGACAAGTGACCATTGACATTCTGAGTCCAGCGGTAGGAGTT CACTGAGGATAGCAGGAAGCCTAGCATCTCAAGCGGAACACTATCTGTAT GGATCTAAGAGTCTTTCAGC/3Phos/

3-piece size marker 2	GCAGAGATTATGTGTAGTTGACATTCTGAGTCCAGCGGTAGGAGTT CACTGAGTAAGTCTCGAAGCCTAGTATCTCAAGCGGAACACTATCTGTATGT GAGGATTCACGTAACAGC/3Phos/
5-piece size marker	GTTAGACAAGTGACCATCGTTGACATTCTGAGTCCAGCGGTAGGA GTTCACTGAGGATAGCAGGAAGCCTAGCATCTCAAGCGGAACACTATCTGT ATGGATCTAAGAGTCTTTCAGCGGGACAGTTCTCAATCTTTGACATTCTGAGT CCAGCGGTAGGAGTTCACTGAGCAGTACATGAAGCCTAGTATCTCAAGCG GAACACTATCTGTATGATTTATCCGTTCTGTGAGCGCAGAGATTATGTGTAGT TGACATTCTGAGTCCAGCGGTAGGAGTTCACTGAGTAAGTCTCGAAGCCTA GTATCTCAAGCGGAACACTATCTGTATGTGAGGATTCACGTAACAGCGGAT ACAATGATAGAGGTTGACATTCTGAGTCCAGCGGTAGGAGTTCACTGAGCA GTACATGAAGCCTAGTATCTCAAGCGGAACACTATCTGTATGTGATTGCCCT CTTCTTAGCGGTTTCGTGGTAGTCATTTGACATTCTGAGTCCAGCGGTAGGA GTTCACTGAGGATAGCAGGAAGCCTAGTATCTCAAGCGGAACACTATCTGT ATGCCTGATAACGATCAAGTTGT
Two-component catalytic strand	CTTCGGATCATAGAGTGGTACGCAGTGTACCAGATCGCTTTCATCCTCAAG TGACTC

Supplementary Table 1: DNA sequences used

**Key**	**Value**
igv	True
sample	None
override_basecaller_cfg	None
max_read_length	None
take_longest_remaining_reads	False
combine_results	False
analyse_unclassified	False
reads_downsampling_size	15000
spoa_max_allowed_read_length	5000
force_spoa_length_threshold	2000
min_read_length	400
medaka_target_depth_per_strand	150
number_depth_windows	100
min_n_reads	40
minimum_mean_depth	30
min_coverage	20
min_read_qual	10
threads	4
primary_alignments_threshold	0.7
spoa_minimum_relative_coverage	0.15
drop_frac_longest_reads	0.05

Supplementary Table 2: Parameters used for variant detection of Oxford Nanopore sequencing data.
